# A GABAergic cell type in the lateral habenula links hypothalamic homeostatic and midbrain motivation circuits with sex steroid signaling

**DOI:** 10.1038/s41398-018-0099-5

**Published:** 2018-02-26

**Authors:** Limei Zhang, Vito S. Hernández, Jerome D. Swinny, Anil K. Verma, Torsten Giesecke, Andrew C. Emery, Kerim Mutig, Luis M. Garcia-Segura, Lee E. Eiden

**Affiliations:** 1 Departmento de Fisiología, Facultad de Medicina, Universidad Nacional Autónoma de México, Mexico City, Mexico; 2 Section on Molecular Neuroscience, National Institute of Mental Health (NIH), Bethesda, USA; 3 Institute for Biomedical and Biomolecular Sciences, School of Pharmacy & Biomedical Sciences, University of Portsmouth, Portsmouth, UK; 4 Department of Anatomy, Charité-Universitätsmedizin Berlin, Berlin, Germany; 5 Instituto Cajal, C.S.I.C., Madrid, Spain; 6 CIBERFES, Instituto de Salud Carlos III, Madrid, Spain

## Abstract

The lateral habenula (LHb) has a key role in integrating a variety of neural circuits associated with reward and aversive behaviors. There is limited information about how the different cell types and neuronal circuits within the LHb coordinate physiological and motivational states. Here, we report a cell type in the medial division of the LHb (LHbM) in male rats that is distinguished by: (1) a molecular signature for GABAergic neurotransmission (Slc32a1/VGAT) and estrogen receptor (Esr1/ERα) expression, at both mRNA and protein levels, as well as the mRNA for vesicular glutamate transporter Slc17a6/VGLUT2, which we term the GABAergic estrogen-receptive neuron (GERN); (2) its axonal projection patterns, identified by in vivo juxtacellular labeling, to both local LHb and to midbrain modulatory systems; and (3) its somatic expression of receptors for vasopressin, serotonin and dopamine, and mRNA for orexin receptor 2. This cell type is anatomically located to receive afferents from midbrain reward (dopamine and serotonin) and hypothalamic water and energy homeostasis (vasopressin and orexin) circuits. These afferents shared the expression of estrogen synthase (aromatase) and VGLUT2, both in their somata and axon terminals. We demonstrate dynamic changes in LHbM VGAT+ cell density, dependent upon gonadal functional status, that closely correlate with motivational behavior in response to predator and forced swim stressors. The findings suggest that the homeostasis and reward-related glutamatergic convergent projecting pathways to LHbMC employ a localized neurosteroid signaling mechanism via axonal expression of aromatase, to act as a switch for GERN excitation/inhibition output prevalence, influencing depressive or motivated behavior.

## Introduction

The habenulae are paired structures located at the dorso-caudal part of the diencephalon and are typically described as having medial and lateral subregions (MHb and LHb). The LHb is notable for receiving inputs from the basal ganglia and forebrain limbic system and projecting broadly to dopaminergic (DA) and serotonergic (5-HT) neurons in the midbrain^1–3^. These midbrain aminergic systems are widely recognized as key components for reward and benefit evaluation processing circuitries^4–7^. Recently, reciprocal inputs from midbrain ventral tegmental area (VTA)^8,9^ and dorsal raphe nucleus (DRN)^10^ have also been observed.

Experimental stimulation of the LHb inhibits midbrain DA and 5-HT neuronal firing (see refs.^3,11,12^). Behaviorally, global activation of LHb has been associated with negative reward prediction error. That is, when an animal receives a reward that is less than expected or receives aversive outcomes greater than expected, there is an increased tendency of cells in the LHb to fire, and the individual will perceive the world in a systematically negative way, as manifested by psychomotor deficiency^6,13^. In vivo electrophysiological recording in primates has provided an elegant demonstration of this conclusion^14^.

Elevated deoxyglucose metabolism has been observed in LHb across animal models of depression^15^. LHb lesion results in reduced depressive behaviors and increased dopamine and 5-HT turnover in the midbrain of rats subjected to chronic stress^16–18^. Clinical findings have shown abnormalities in habenula in depression^19,20^. Disruption of LHb firing by deep brain stimulation (DBS) produces remission of severe depression, while shutting off DBS correlated with patient’s relapse, and re-installation of DBS remitted depression again^21^. All of these findings call attention to the LHb as a potential locus for dysfunction in human neuropsychiatric disease, including the negative symptoms of depression, particularly if the intrinsic circuit-based functional connectivity and modulatory mechanisms governing LHb synaptic and circuit plasticity can be better understood.

The LHb contains widely and densely distributed vesicular glutamate transporter 2- and 3- (VGluT2 and VGluT3)-expressing glutamatergic neurons^22,23^ and many of them also express membrane GABA transporters 1 and 4 (mGAT1 and mGAT4)^24^. There is also a prominent presence of neuropeptides, especially in the medio-central subdivision of the lateral habenula (LHbMC)^25–34^. A small number of selectively distributed neurons in LHb express typical GABAergic neuron markers GABA and GAD-65/67^34–36^. Consistent with the notion of intrinsic GABAergic neurons in LHb, a careful anatomical study using Golgi–Kopsch silver impregnation method, done by Iwahori 40 years ago, unambiguously identified a neuron type “IV” as small cells with short axons, suggesting the existence of a neural circuitry intrinsic to the LHb^37^. However, the existence of functional GABAergic neurons intrinsic to LHb is currently a topic of debate since a complete description of GABAergic phenotype of a single population of neurons in LHb, including the existence of the GABA vesicular transporter VGAT, does not yet exist^38^.

In addition to its neurotransmitter complexity the LHb also features a rich expression, and selective localization, of estrogen receptor-alpha (ERα)^39,40^, suggesting regulation of LHb function by sex steroids. Sex steroid effects reported have focused on female rat sexual receptivity and maternal behavior^40^, however a recent paper reported that in an ex vivo preparation, estradiol suppressed global neuronal activities in the LHb region of male rats^41^.

Previously, we reported that the medial division of lateral habenula (LHbM) in male rat hosts sparsely distributed GABAergic neurons that are particularly active during response to homeostatic challenge. Local activation of these neurons is linked to suppression of global LHb activity, suggesting that they are inhibitory within LHb, and, potentially, functionally promote motivational behavior^34^. In the current study, we adopted a combinatorial approach to further investigate this intrinsic LHb GABAergic system, its inputs and outputs, its dependence on hormonal conditions and the consequences of in vivo homeostatic manipulation of LHb synapses on modulation of motivated behaviors in the rat.

## Materials and methods

### Animals

One hundred and fifteen male Wistar rats from a local animal breeding facility were used in this study. All procedures were approved by the Research and Ethics Committee of the Faculty of Medicine, Universidad Nacional Autónoma de México (IDs CIEFM-085-2013 and CIEFM-062-2016), in accordance with the principles espoused in the Handbook for the Use of Animals in Neuroscience Research (Society for Neuroscience. Washington, DC 1991 and as updated periodically). Four groups were employed: sexually active (SA, 300–500 g, b.w., housed under standardized conditions, but each housed with two females rats for three-day periods over a period of 12 weeks with periodic harem changes; *n* = 20); sexually inactive (SI, 300–450 g, housed with male rats, three per cage, standard conditions of animal house, *n* = 100), gonadectomized rats, housed as for SI (Gnx, *n* = 40. see section “Gonadectomy (Gnx) and hormone replacement therapy (HRT)”) and Gnx treated with testosterone, housed as for Gnx (Gnx-HRT, *n* = 10).

### Chemicals

Chemicals and reagents were obtained from Sigma-Aldrich, St. Louis, MO, USA, if not indicated otherwise. Primary antibodies used in this study were against vasopressin (rabbit anti-AVP, Peninsula-Bachem American, Inc. USA, CA, T-4563, 1:4000), vasopressin (rabbit anti-AVP, gift from R.M. Buijs^42^, 1:2000), tyrosine hydroxylase (sheep anti-TH, EMD Millipore Corporation, MA, AB-1542, 1:4000), serotonin transporter (goat anti-SerT, Santa Cruz Biotechnology, CA, SC-1458, 1:2000), hypocretin/orexin (rabbit anti-OR, gift from A. van del Pol^43^), vesicular glutamate transporter 2 (guinea pig anti-VGluT2, Frontier Institute, Co., Japan, gp-AF240-1, 1:1000), vesicular inhibitory amino acid transporter (rabbit anti-VGAT/VIAAT, provided by L. E. Eiden^44^, 1:1000), GABA (mouse anti-GABA, Sigma-Aldrich Co. MO, A0310, 1:1000), glutamic acid decarboxylase 65 kDa isoform (mouse anti-GAD-65, EMD Millipore Co. MA, MAB351, 1:2000), glutamic acid decarboxylase 67 kDa isoform (mouse anti-GAD-67, EMD Millipore Co. MA, MAB5406, 1:2000), parvalbumin (mouse anti-PV, Swant, Switzerland, Cat. 235, 1:5000), P450 Aromatase (rabbit anti-ARO, provided by L. M. García-Segura^45^, 1:2000), P450 Aromatase (rabbit anti-ARO, Abcam, Cambrdge, UK, AB18995, 1:2000), P450 Aromatase (mouse anti-ARO, Acris, SM2222P, 1:200), estrogen receptor-alpha (rabbit anti-ERα, Santa Cruz, CA, SC542, 1:2000), androgen receptor (rabbit anti-AR, Santa Cruz, CA, SC816, 1:2000), dopamine receptor 5 (rabbit anti-D5R, also called D1Rb, Alomone, Israel, 1:1000), serotonin receptor (mouse anti-5-HTR2a, BD pharmingen, Cat. 556326, 1:200), vasopressin receptor 1a (rabbit anti-V1a, provided by K. Mutig and T. Giesecke, see SI method and SI Fig. 3 for details), and green fluorescent protein (mouse anti-GFP, Abcam, Cambridge, UK, Ab291-50, 1:500). See SI Table 1 for detailed information.

### Juxtacellular labeling

For this study, juxtacellular recording and labeling was performed in 48 male Wistar rats (300 g) according to previous protocols^33,34,46–48^. The induction of anesthesia was achieved using 4% v/v isoflurane (Sofloran Vet, Pisa, Mexico) in O_2_ and maintained with urethane (1.3 g/kg, i.p.; ethyl carbamate; Sigma) and supplemental doses of ketamine (30 mg/kg, i.p.; Anesteket, Pisa, Mexico) and xylazine (3 mg/kg, i.p.; Procin, Pisa, Mexico). Wound margins were infiltrated with local anesthetic (lidocaine, Pisa, Mexico). A stereotaxic frame (David Kopf Instruments, CA) was used to fix the animal in place and a homeothermic heating device (Harvard Apparatus) was used to maintain core temperature at 36 ± 0.5 °C. Craniotomy was performed around the coordinates: −3.5 mm posterior from Bregma and 0.5 mm lateral. See the supplemental material for juxtacellular labeling procedure details. After the procedure, the rats were maintained at 35 °C during 4–6 h before perfusion. Brain tissue was subsequently processed as described in supplemental material. Sections containing well-labeled somata, revealed by streptavin-Alexa488 reaction, and with observable axon-branching patterns indicative of biotin diffusion at least beyond the cell soma were further processed for VGAT/VGLUT2 immunoreaction and ERα immunofluoroscence reaction with corresponding secondary antibodies. Three neurons from three different rats met inclusion criteria for this study, which included visualization of axon-like branching within the LHb, allowing for their post-hoc molecular and morphological characterization. Exclusion criteria included limited labeling, multiple cell labeling, lack of internally branched profiles, or anatomical localization outside of LHbM. For more details, see the SI Experimental Procedures.

### Immunohistochemistry

Immunohistochemistry was carried out using a standard free-floating method as described previously^34^. Unless specified otherwise, we used SA rats (300–400 g, b.w., *N* = 5) for IHC. In the experiments where the effect of gonadal steroids was evaluated, we used Gnx and Gnx-HRT rats. See SI Table 1 for detailed primary antibody information.

### Fluoro-gold retrograde tracing

Fluoro-gold retrograde tracing was performed according to previously published protocols^49^. Ten male Wistar rats (b.w. 300 g) were used. See SI Experimental Procedures.

### RNAscope In Situ Hybridization (ISH) assays

The ISH probes for rat Slc32a1(mRNA encoding VGAT), Slc32a1-C3 (mRNA encoding VGAT in channel 2), Slc17a6 (mRNA encoding VGLUT2), Slc17a6-C2 (mRNA encoding VGLUT2 in channel 2), Gad1 (mRNA encoding GAD-67), Gad2 (mRNA encoding GAD-65), Esr1 (mRNA encoding ERα), Hcrtr2 (mRNA encoding orexin receptor 2) were designed and provided by Advanced Cell Diagnostics (Hayward, CA). All steps were performed following RNAscope protocols for RNAscope Fluorescent Multiplex Assay, 2.5 HD, Duplex Assay and 2.5 HD Assay-Brown for rat brain fresh frozen tissue. For VGAT mRNA (Slc32a1) expressing neuron density assessment, we cryosectioned four brains per group (*N* = 16) and kept five series (A–E) of habenula per sample. Sections (12 μm thick) located at the same position of each series were continued sections. Hence, one series per each brain was fixed and stained with hematoxylin and served as anatomical reference for ISH section selection. Sections containing two habenulae (left and right as habenular asymmetry has been recognized^50,51^) around Br. −3.72 mm from four rats (*n* = 8) were used. Complete experimental methods are described in SI Experimental Procedures.

### Gonadectomy (Gnx) and hormone replacement therapy (HRT)

Juvenile male rats of post-natal day 35 were used (*N* = 40). Under anesthesia with ketamine (100 mg/kg, IP) and xylazine (10 mg/kg, IP, Procin, Pisa, Mexico), a small surgical incision was made in the center of the scrotum. The testicles and spermatic cord were exposed through the surgical wound, then the spermatic cord was cauterized and the testicles removed. The incision was closed with nylon 3-0 sutures and rats treated with ketorolac and ceftriaxone during the post-operative period.

For HRT, after 60 days of Gnx, 10 subjects received monthly s.c. injections of Sustanon (dose: 250 mg/kg body weight). Sustanon 250 is a long-acting mixture of testosterone esters: testosterone propionate (20%), testosterone phenylpropionate (40%), and testosterone isocaproate (40%) (Organon Mexicana, CdMx, Mexico). Rats were housed two per cage. See SI Experimental Procedures for further details.

### Live cat exposure, forced swimming test, and behavioral scoring

The experiments were performed according to a previously published protocol^34^ (*n* = 10). See SI Experimental Procedures.

### Data analysis

Quantitative results were expressed as mean ± SEM. Groups were tested for normality with a D’Agostino and Pearson’s test. Differences between paired groups were calculated by Student’s two-tailed *t*-test. Multiple group comparisons were performed using Bonferroni post hoc test after ordinary one-way analysis of variance (ANOVA), specified in the “Result” section for each experiment. Post-hoc differences were considered statistically significant at a value *p* < 0.05 (*p < 0.05, **p < 0.01, ***p < 0.001).

## Results

### The LHbMC hosts a cell type expressing nuclear ERα and VGAT at axon terminals, with dual local circuit and long-range projection patterns

**Fig. 1:**
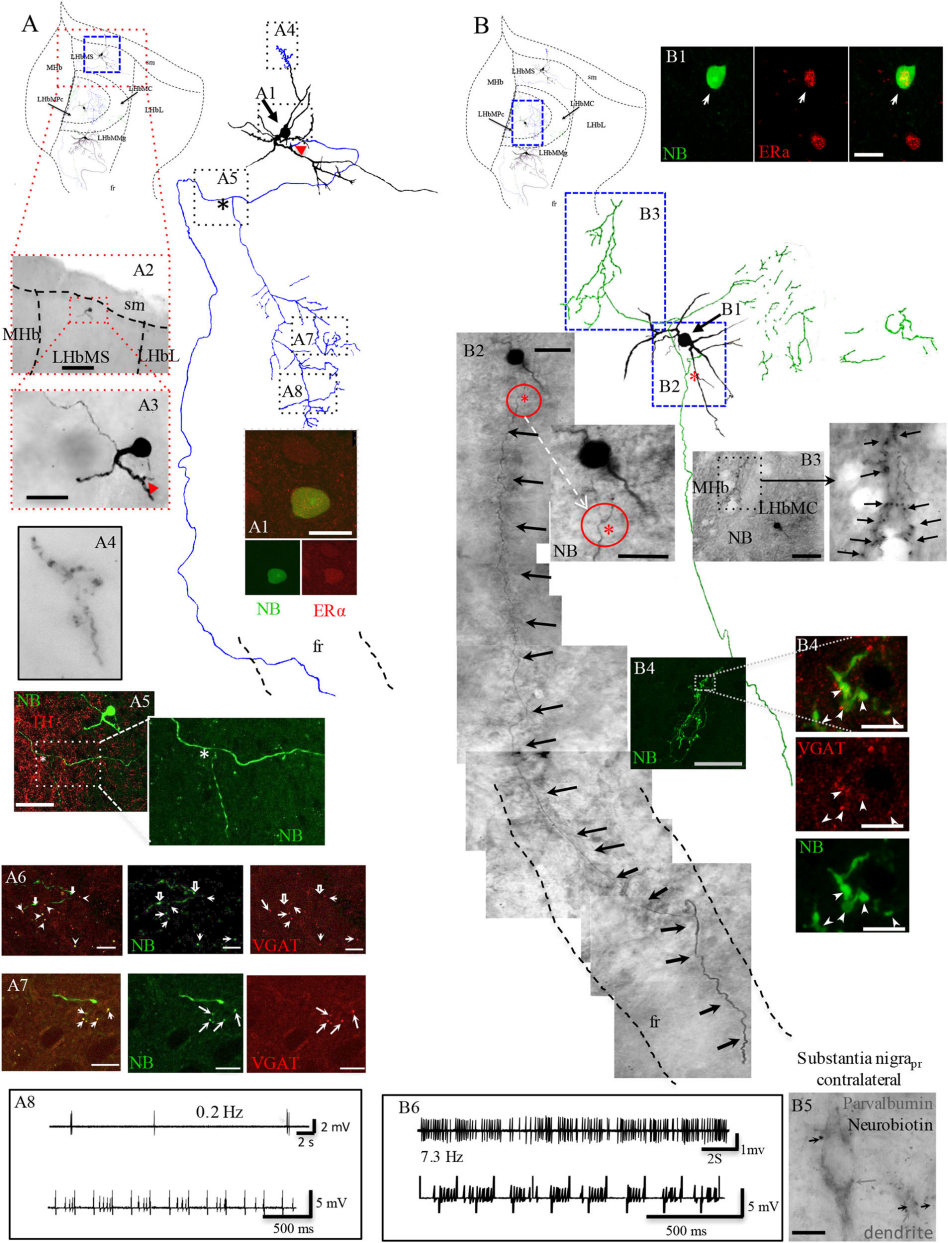
A novel type of GABAergic neuron with bi-functional output, was identified using in vivo juxtacellular single-cell labeling, immunostaining, and anatomical reconstruction methods. Three cells are reported in this study, one per each nucleus of the medial division of the lateral habenula (the superior, LHbMS, the central, LHbMC, and the marginal, LHbMMg) are depicted in one schematic coronal view of the habenula aiming to give a general idea about their spatial relationship with the region and among them, although they were from three different rats. Camera-lucida reconstruction from serial sections of the neuronal somata, dendrites, and axons were superimposed manually in a 2-dimension (2D) projection drawing from a coronal view of the rat habenula. The soma and dendrites were represented in black and axonal segments were color-coded as blue for cell A (located in the LHbMS), and green for cell B (located in the LHbMC) (see the third cell in SI Fig. 1). A_1_: The soma of the cell A is immunopositive to ERα. A_2_, A_3_: photomicrographs of labeled soma and proximal dendrites revealed by avidin-biotin-peroxidase diaminobenzidine reaction, at low and high magnifications, red arrowhead in A_3_ shows the emergence site of the main axon. A_4_: photomicrograph of a rare axonal terminal-like arborization observed at the squared region in the reconstruction. A_5_: the main axon emitted a single collateral that coursed ventrally and branched in the medial central region of the LHb (the branching point is indicated by an asterisk, also shown in the inset and in the reconstruction). Note in the reconstruction that the projecting axon entered to the fr and was found in further caudal sections. A_6_, A_7_: neurobiotin/VGAT double labeling at axon terminals (white solid arrows). Note that some neurobiotin-labeled axonal VGAT (hollow white arrows). A_8_: upper traces. extracellular recording of low spontaneous firing pattern and lower trace shows that when low intensity (<10 nA) positive current pulses were injected by way of the microelectrode, the neuron firing pattern was modulated, a requirement to yield a successful labeling. Scale bars: A_1_: 25 µm; A_2_: 500 µm; A_3_: 50 µm; A_5_: 100 µm; A_7_, A_8_: 10 µm. B_1_: Immunohistochemical detection of ERα expressed in the neurobiotin-labeled cell. B_2_: a compound photomicrograph made up by photomicrographs taken from 11 consecutive sections showing the neurobiotin-labeled soma and the main axon projecting to the fr. In a proximal point of the main axon, an axon-collateral was emitted (indicated by a red asterisk and a circle). Inset shows a higher magnification photomicrograph of the collateral origin point. B_3_**:** shows the intrahabenular branched axon segments and ATs labeled with neurobiotin (NB). B_4_: confocal images show the GABAergic nature of this cell (ATs immunopositive to VGAT). B_5_: Axon terminals found in caudal sections at the level of sustantia nigra pars reticulata, in close apposition with parvalbumin-expressing dendrites. B_6_: upper traces are extracellular recording of spontaneous firing patterns (7.3 Hz before electrical modulation applied for juxtacellular labeling purpose, lower trace). Scale bars: 20 µm except B_3_: 100 µm and B_5_: 10 µm

We previously described the existence of three in vivo juxtacellularly labeled GABA/GAD-positive neurons in LHbMC^34^. In this study, we sought a more complete characterization of this cell type via identification and reconstruction after in vivo juxtacellular labeling. Three more cells were successfully labeled and internally branched axons and axon terminal (ATs) were filled with Neurobiotin (Fig. 1; SI Figs. 1 and 2). We found that all three labeled neurons expressed nuclear immunoreactivity for ERα (Fig. 1a, A_1_ and b, B_1_; SI Fig. C1), with their ATs immunopositive for VGAT (Fig. 1a, A_6_–A_7_ and b, B_4_; SI Fig. 1, C6), but not for VGLUT2 (data not shown).

All these neurons had local as well as long-range axonal collaterals. Indeed, for one cell, the labeled axon was traced to the midbrain at which point one branch crossed the midline and branched inside the substantia nigra pars reticulata (SNpr), with axon terminals making appositions onto parvalbumin IR somata and dendrites (SI Fig. 2). Note that additional branches of this neuron into more caudal extensions of fasciculus retroflexus (fr) may exist, with Neurobiotin visualization impeded by white matter of this conduction system^52,53^. This confirms the presence of an LHb GABAergic cell type distinguished by expression of ERα and a likely a role both within the LHb and at midbrain centers. In the following we call these GABAergic estrogen-receptive neurons (GERNs) for convenience of referral.

### High-resolution anatomical and molecular interrogation of LHbM GERNs

**Fig. 2:**
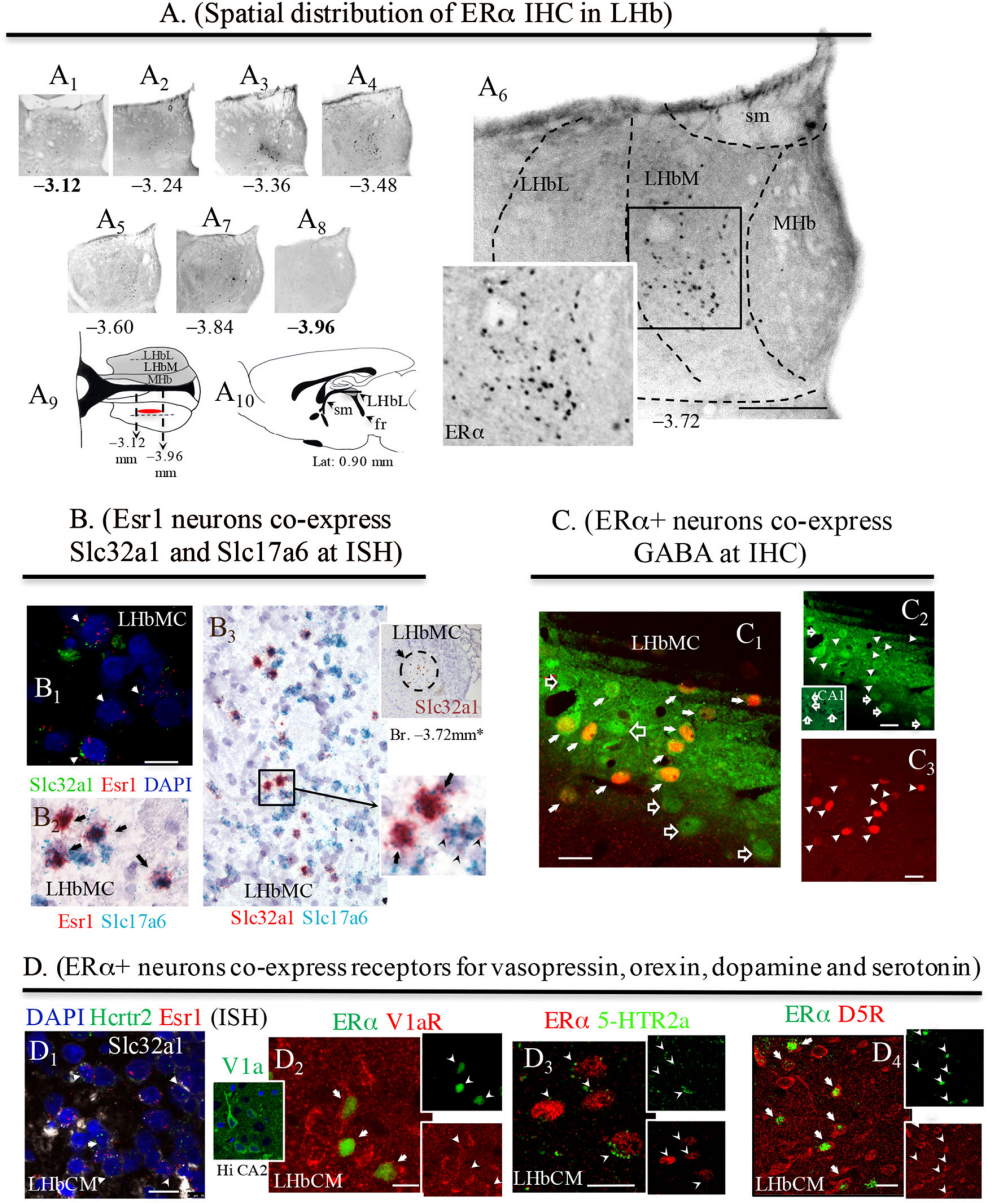
GABAergic estrogen-receptive neurons (GERNs) localization, mRNA expression by ISH and somatic receptor expression by IHC. **a** Serial coronal sections showing the ERα immunolabelling at the Bregma rostro-caudal coordinates (numbers in mm under the photomicrographs). Boxed area in A_6_ at higher magnification showing the exclusive nuclear labeling pattern. The bold numbered levels (A_1_ and A_8_), were chosen to show that no positive labeling was found in either rostral or caudal directions. A_9_: a horizontal view of the distribution of estrogen-receptive cells was symbolized by the red oval. A_10_: sagittal view of rat brain atlas, modified from Paxinos & Watson^86^, at lat. 0.90 mm, where lateral habenula (LHb) is symbolized with a gray shade and A9 plane was symbolized with a horizontal line. **b** In situ hybridization using multiple RNAscope methods. B_1_: multiplex fluorescence method, Esr1, gene that encodes ERα (red punctuated labeling) co-expressed with Slc32a1, gene that encodes VGAT (green punctuated labeling); arrows indicate the double-labeled cells; B_2_: duplex method, Esr1 (red punctuated labeling) co-localization with Slc17a6, gene that encodes VGLUT2 (green punctuated labeling); arrows indicate the double-labeled cells; B_3_: with duplex method, Slc32a1 encoding VGAT (red punctuated labeling) shows complete co-localization with Slc17a6 encoding VGLUT2 (green punctuated labeling); arrows indicate the double-labeled cells. Inset of B_3_, Slc32a1 expression in a sexually active (SA) rat LHb, Br. −3.72 mm (brown labeling, single chromogenic-Brown method-RNAscope). *Note the similarity with ERα expression in A_6_. **c** Indirect immunohistochemistry showing the GABAergic nature of the ERα+ neurons (red) in a SA rat brain. The GABA antibody (green, Sigma, A0310) produced characteristic surface labeling (C_1_, C_2_: the strip-like image was produced by Vibratome slicing leaving the brain section with an uneven surface). **d** ERα+ neurons co-express receptor/receptor subtypes for vasopressin, orexin, dopamine, and serotonin. D_1_: In situ hybridization using RNAscope-multiplex method targeting Esr1 (red dots), Slc32a1 (white dots) and Hcrtr2, gene encodes the orexin receptor 2 (green dots). D_2_, D_3_: Indirect immunofluorescence reactions, showing that the ERα-IR cells co-expressed vasopressin receptor V1a (inset showing the V1a antibody labeling pattern in temporal hippocampus CA2 cells body layer. See also SI Fig. 6 for more information about this antibody), dopamine receptor D5R (also called D1Rb) and serotonin receptor 5-HTr2a, respectively. Scale bars: A_5_ and **b**: 500 µm and rest: 10 µm

The population which selectively expressed ERα-IR was restricted to specific anatomical coordinates of within the LHbM (Fig. 2a): we did not detect ERα-positive cells in either rostral or caudal portions of LHb (Fig. 2a, A_1_ and A_8_). With RNAscope ISH we found that neurons in the LMbMC co-expressed mRNA for ERα (Fig. 2b, B_1_, red punctate labeling) and Slc32a1, mRNA encoding VGAT (green punctate labeling, arrows indicate double-labeled cells), as well as Slc17a6, encoding VGLUT2 (Fig. 2b, B_2_, green punctate labeling for Slc17a6; arrows indicate double-labeled cells). Unexpectedly, every single Slc32a1-positive neuron was also positive for Slc17a6 (Fig. 2b).

The Slc32a1 signal was restricted to the central medial nucleus LHbMC of rat lateral habenula (circumscribed region of Fig. 2 upper inset of panel B_3_, RNAscope chromogenic-Brown method). At the immunohistochemical level, the majority (68%) of GABA-expressing neurons were immunopositive for ERα (Fig. 2c). Furthermore, these Esr1+/Slc32a1+ cells also co-expressed Hcrtr2 (mRNA enconding hypocretin/orexin receptor 2) (Fig. 2d, D_1_), and were immunopositive for V1aR (Fig. 2d, D_2_), for HTR2A (Fig. 2d, D_3_), and for D5R (Fig. 2d, D_4_).

### Projections containing AVP, orexin, dopamine, and serotonin to LHbM exhibited a common molecular signature of aromatase and VGLUT2 expression

**Fig. 3:**
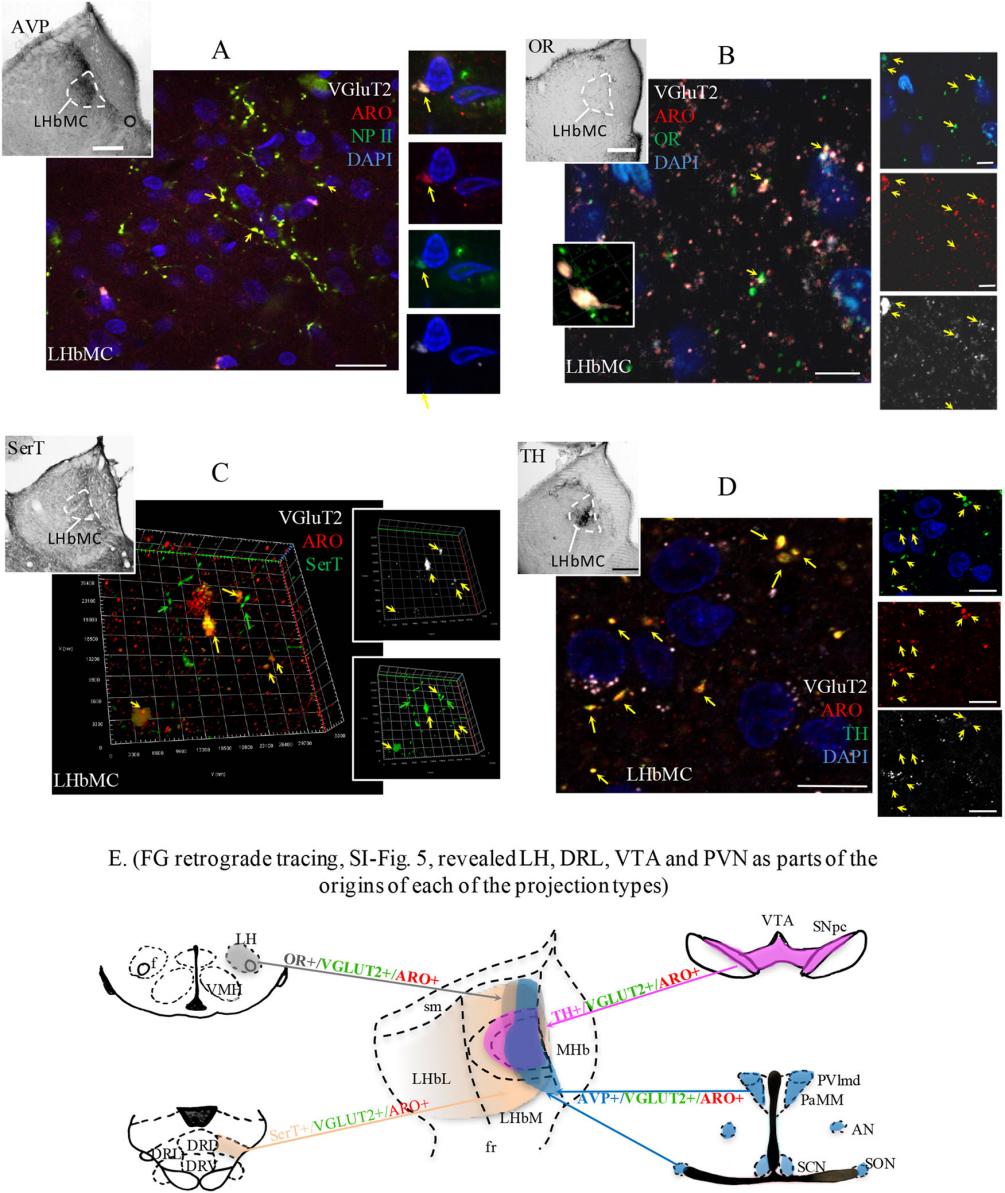
Presence of convergent inputs from vasopressinergic, orexinergic, dopaminergic and serotoninergic pathways expressing VGluT2 and P450 aromatase in LHbM where the GERNs cells were located. **a**–**d** Confocal images revealed that axon terminals immunopositive for neurophysin II (NPII, **a**), orexin (OR, **b**), serotonin transporter (SerT, **c**), and tyrosine hydroxylase (TH, **d**) contained aromatase (ARO) and vesicular glutamate transporter 2 (VGLUT2) inside the LHbMC. Note in **c**, 3D computer reconstruction of the serial optical slices in *Z*-stack to show that fibers which expressed SerT were of two types: thick SerT+/VGLUT2+/aromatase+ (yellow arrows) and thin SerT+/VGLUT2−/aromatase− profiles (green arrows). Insets for each confocal photomicrograph group show the global fiber distribution patterns with peroxidase immunoreaction against arginine vasopressin (AVP, **a**), orexin (OR, **b**), serotonin transporter (SerT, **c**), and tyrosine hydroxylase (TH, **d**) at the LHbM region. A detailed anatomical distribution from serial coronal sections is depicted in SI Fig. 3. **e** Summary diagram of FG retrograde tracing results presented in SI Fig. 4. The upstream regions identified by FG retrograde tracing experiments are coded by colors: hypothalamic vasopressinergic nuclei in blue; lateral hypothalamic orexinergic cell population in gray; dorsal raphe lateral (DRL) serotonin transporter (SerT) expressing neurons in beige; substantia nigra pars compact (SNpc), and ventral tegmental area (VTA) tyrosine hydroxylase (TH) expressing neurons in pink. The projection distributions of each pathway, in the habenula region, are symbolized with the corresponding color patches. The beige gradient filling symbolizes the predominant distribution pattern of SerT+ fibers observed (for details see SI Fig. 3D). The four pathways to habenula (color-coded arrows) shared a common feature of co-expression of VGLUT2 (symbolized in green) and estrogen synthase/P450 aromatase (symbolized in red). Scale bars: **a**–**d** 500 µm and **e**–**h** 10 µm

We have identified LHbM as a prime region of convergent input of hypothalamic vasopressin (AVP) and orexin, and midbrain dopamine and serotonin systems by immunohistochemistry in combination with fluoro-gold retrograde tracing (SI Figs. 4 and 5). In light of the selective location of GERNs in the LHbM, and the apparent absence of estrogen synthetic capacity by cells of LHb, we asked whether the pre-synaptic inputs to these cells might produce estrogen. Using IHC and confocal microcopy we found that the four types of inputs to LHbMC were immunopositive for VGLUT2 and aromatase (Fig. 3a–d). The hypothalamic vasopressinergic paraventricular, supraoptic and suprachiasmatic nuclei (PVN, SON, and SCN, respectively) and orexinergic lateral hypothalamic area (LH) were identified as hypothalamic inputs to LHbM, as were the dopaminergic substantia nigra (SN) and ventral tegmental area (VTA), and the serotonergic dorsal raphe lateral (DRL) nucleus, as the midbrain inputs to LHbM (SI Fig. 5B–D). Results pertaining to aminergic and peptidergic projections to LHbM are summarized diagrammatically in Fig. 3e. The clear demonstration of these inputs to the LHbM is consistent with the current literature (see a recent review^36^) but does not exclude convergence of other types of projections to the LHbM.

### Hypothalamic peptidergic and midbrain aminergic inputs to LHbM with a shared sex steroid-responsive phenotype

**Fig. 4:**
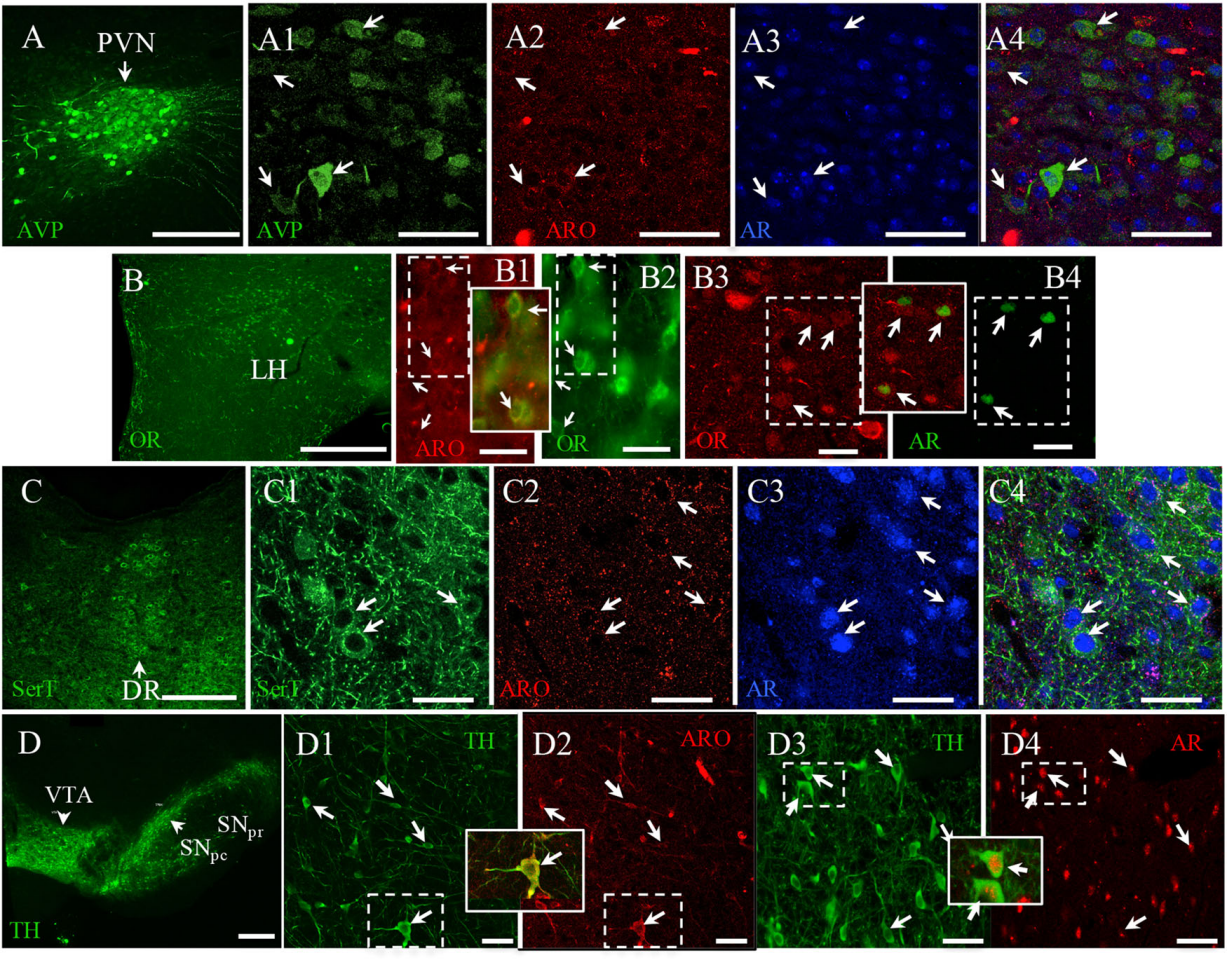
Androgen receptor (AR) and P450 aromatase (ARO) expression in PVN, LH, VTA/SNpc and DRL, AVP+, OR+, TH+ and SerT+ neurons, respectively. The series of **a**–**d** show the confocal images for co-expression of AR, aromatase and AVP (PVN, **a**), orexin (LH, **b**), SerT (DR, **c**) and TH (VTA, **d**). Examples of co-expressed cells are indicated with arrows. Scale bars: **a** 500 µm; A_1_–A_4_: 50 µm; **b** 500 µm; B_1_–B_4_: 20 µm; **c** 500 µm; C_1_–C_4_ 25 µm; **d** 1 mm; D_1_–D_4_ 50 µm

We performed immunofluorescence experiments to evaluate the co-localization of androgen receptor (AR) and aromatase (ARO) in cells of hypothalamic AVP+ and OR+ nuclei and in midbrain cells of SerT+ and TH+ nuclei. Most of the amine-positive or peptide-positive neuronal nuclei were also immunopositive for AR, with a smaller but substantial number displaying aromatase-positive cytoplasm (Fig. 4).

### Regulation of midbrain to GERN and hypothalamo to GERN circuits, and aversive behavioral responses, by hormonal status

**Fig. 5:**
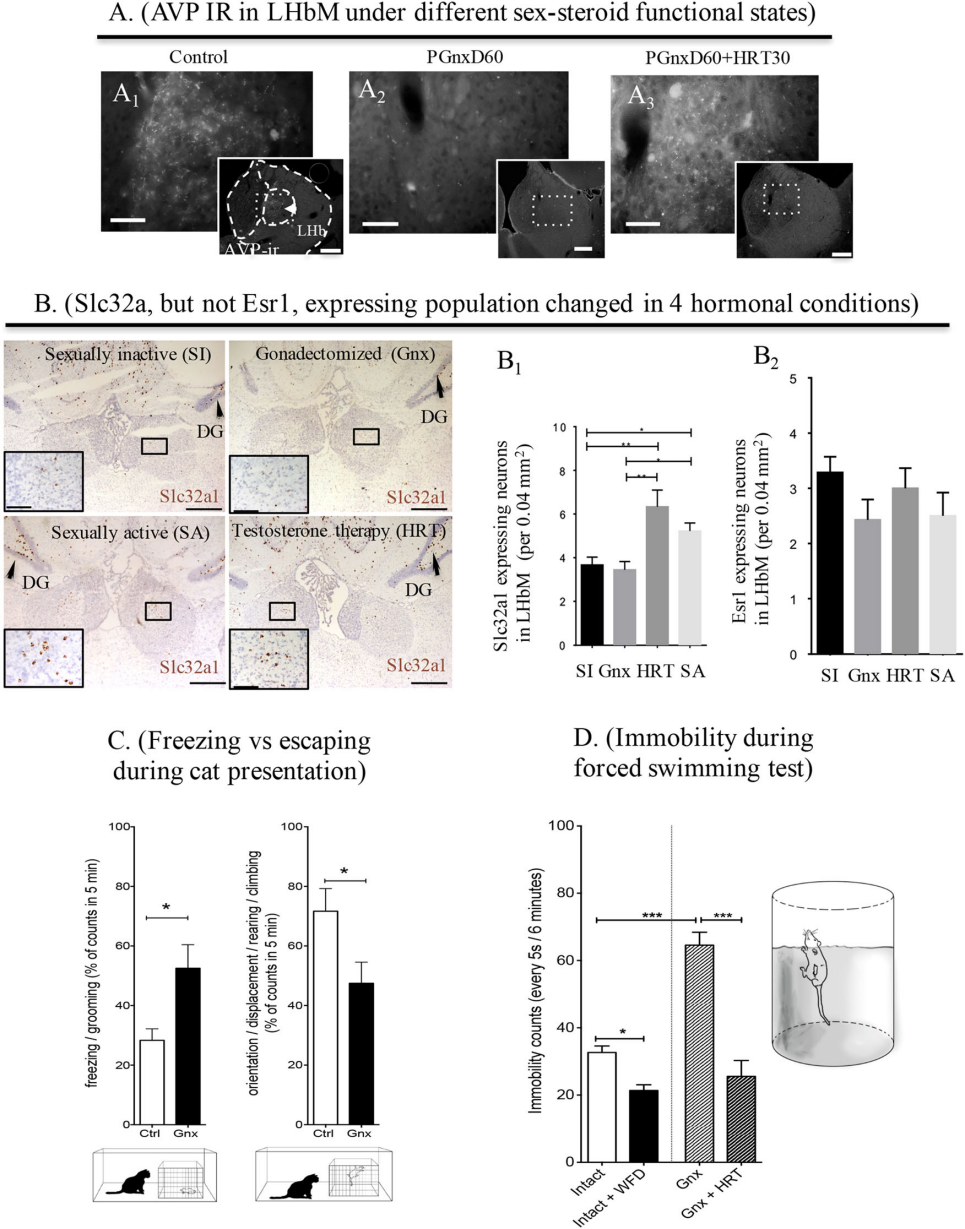
Hormonal conditions affect GERN-LHbM input composition and cell density: correlation with depressive vs motivated behaviors. **a** Gonadectomy in male rats produced a remarkable reduction of AVP immunoreactivity in lateral habenula medial (LHbM) that is restored with hormone (testosterone) replacement therapy (HRT). A_1_: Control; A_2_: AVP-IHC performed 60 days after gonadectomy (PGnxD60), and A_3_: 60 days after gonadectomy animals received hormone replacement therapy for 30 days (PGnxD60+HRT30). **b** Photomicrographs showing in situ hybridization experiment using RNAscope 2.5 HD Assay-BROWN method. B_1_: Slc32a1 cell density changed in the LHbM under the four hormonal conditions. HRT (**p < 0.01) and sexual activity (*p < 0.05) significantly increased the number of Slc32a1 neurons in LHbM. B_2_: No significant differences were found between all groups SI and GNX rats with respect to the Esr1 expression. **c** Upon cat exposure, rats expressed innate fear-related passive (freezing), and active (rearing, climbing, and displacement) behaviors: rats from 60 days post gonadectomy group (Gnx) showed significant increase of freezing counts (*p = 0.0203) and reduced number of climbing and rearing behaviors (*p = 0.0421). **d** Quantification of behavioral despair assessed using forced swimming test (FST). Gnx significantly increased the immobility counts in the FST (***p < 0.001). Gnx rats that received HRT recovered to levels comparable to control animals. By contrast, water and food deprivation had the opposite effect, significantly decreasing the immobility counts compared to control (*p < 0.05)

To assess involvement of testosterone in these circuits, we employed two sets of experimental conditions. GERN afferents were compared in intact and gonadectomized (Gnx) males. Gonadectomy produced a remarkable reduction of AVP-IR in the SON and PVN nuclei (SI Fig. 6A–F), as well as a reduction in the percentage of aromatase/AVP double-labeled cells (SI Fig. 6G). In addition, AVP-IR fibers almost disappeared completely in the LHbM (Fig. 5a, A_1_ vs A_2_). Our results are in accordance with previous studies showing AVP system hypofunction as a result of decreased gonadal steroid levels^54,55^. Thirty days after the onset of testosterone replacement therapy (HRT) (PGnxD60+ HRT30), the AVP-labeling pattern was restored (Fig. 5a, A_3_). The orexinergic, dopaminergic, and serotoninergic systems, however, were not noticeably affected by gonadectomy (data not shown), albeit fully processed orexin-, and DA- and 5-HT-positive terminals themselves, were not directly assessed in our experiments.

In a second set of experiments, the effects of hormonal conditions on the Slc32a1-positive cell density in LHbMC was examined using RNAscope methods. Sexual activity was also examined as a variable, since sexual activity with unfamiliar female subjects has been shown to increase the levels of testosterone in males^56,57^. We found significant differences between groups in the number of Slc32a1+ neurons. (one-way ANOVA ***p = 0.0003). The number of Slc32a1-positive cells in LHbMC was significantly greater in SA compared to SI male rats (*p < 0.05), and Slc32a1 expression was also markedly increased by HRT in gonadectomized male rats (**p < 0.01, Fig. 5b). The effect of hormone replacement therapy (HRT, for 30 days in Gnx male rats), or sexual activity, on the number of Slc32a1*-* or Esr1-expressing neurons in the LHbM was quantified (Fig. 5b, B_1_ and B_2_). HRT and sexual activity significantly increased the number of Slc32a1 neurons in LHbM, with no significant differences found between SI and Gnx rats. With respect to the Esr1 expression after Gnx, HRT, or sexual activity, no significant differences were found.

Finally, we investigated possible implications of the above mentioned manipulation on depressive vs motivational behaviors in two simple tests, placing the rats in psychological and physical life-threatening conditions: (i) assessing the innate fear processing using the exposure to a live cat^34,58^, and (ii) assessing behavioral despair using a modified version of the forced swim test (FST)^59,60^. Negative and positive motivational valence representations were correlated with freezing vs. rearing/climbing/displacement during cat exposure and immobility vs climbing during FST. As the major neurochemical effects of manipulation of testosterone levels was on GERNs themselves, and on pre-synaptic vasopressinergic inputs to the LHb, and as we have previously observed that osmotic hypothalamic magnocellular AVP upregulation suppressed the LHb functional output and promoted escape behaviors during predator exposure and behavioral despair test (FST), we evaluated intact undisturbed rats, relative to rats with water and food deprivation for 24 h (WFD). These conditions were compared with gonadectomized (Gnx) rats, and Gnx + HRT rats.

The effects of Gnx on the passive stress coping strategies (freezing) or active stress coping strategies (rearing/climbing/displacement), displayed when exposed to a live predator, are shown in Fig. 5c. The Gnx group showed an increase (52.54 ± 7.092 vs control 28.33 ± 3.145 counts, *n* = 6, *p* < 0.05) in the passive (freezing, grooming) behavior and a decrease (47.46 ± 7.092 counts vs control: 71.67 ± 7.600 counts, *p* < 0.05) in active escape (climbing, rearing, displacement, orientation) behaviors.

The effect of Gnx combined with hormone replacement therapy (HRT) or water and food deprivation (WFD) on behavioral despair was quantified as the number of immobility episodes in the forced swimming test (FST) (Fig. 5d). Differences between means were found statistically significant by one-way ANOVA (F_(3, 23)_ = 44, ***p < 0.0001). Gnx significantly increased the immobility counts in the FST (33 + 1.8 vs. 65 + 3.8 counts in control and GNX, respectively, ***p < 0.001), whereas castrated (Gnx) rats that received HRT recovered to levels comparable to control animals (control: 33 + 1.8 counts vs. HRT: 25 + 4.7 counts, *p* > 0.05). Water and food deprivation, in turn, significantly decreased the immobility counts relative to control.

## Discussion

Enhanced excitatory input to LHb is generally associated with global activation of this nucleus, leading to inhibition of the connected midbrain monoaminergic systems, driving aversion and psychomotor deficiency^6^. This dogma has been supported by extensive studied using a variety of experimental strategies, including short-term circuit manipulation techniques^61,62^. However, here, by studying the mid-term and long-term hormones influences on LHb circuitry organization and the subsequent behavioral modifications, in male rats, we obtained data that challenge the completeness of the above notion.

Previously, we established the existence of GABAergic interneurons inside the LHb. Here, we show key properties of these same neurons, using the linking technology of juxtacellular labeling in vivo, and the use of a new antibody against the vesicular GABA transporter (VGAT) that unambiguously identify these cells as GABAergic (as GAD is no longer considered such a definitive marker for functionally GABAergic cells), we identify them as relevant to peptidergic/neurosteroid synaptic transmission with implications to gonadal/neurosteroid modulation of behavior. The projection axons of these GERN cells enter the fasciculus retroflexus and at least one cell sent projections to the region of the tail of VTA (RMTg) and the medial part of substantial nigra pars reticulata, making close contact to parvalbumin positive neurons. These characteristics establish the potential novelty and importance of GERN cells in LHb circuitry, and we then set out to identify the impingents upon these cells, and their potential role in homeostatic and hormonal/sex steroid modulation of motivated behavior.

In this study, using complementary molecular, electrophysiological, anatomical, hormone status manipulation, and behavioral approaches, we have identified four glutamatergic inputs to the LHbM from hypothalamic water and energy homeostasis and midbrain reward/value evaluation circuits, containing vasopressin, orexin, dopamine, and serotonin, respectively, where a discrete estrogen-receptive cell population expressing molecular signatures for both GABA and glutamate neurotransmission were located. Juxtacellularly labeled single VGAT+/ERa+ cells within this region emitted main axons joining the fasciculus retroflexus, as well as locally branching collaterals. Single-labeled neurons lacked light-microscopically visualizable VGLUT2 terminal immunoreactivity, suggesting that their functional neurotransmitter phenotype is likely inhibitory.

Although there have been recent intriguing discoveries that detail glutamate to GABA transmitter switching in habenular inputs^63^, the GABAergic microcircuit intrinsic to the habenula has remained enigmatic. Early morphological studies suggested the existence of interneurons^37^, and GAD activity was only reduced 40% in stria medullaris-lesioned rat, indicating the existence of a source of GABA intrinsic to the habenula^64^. However, evidence for phenotypically patent (i.e., VGAT-positive) GABAergic neurons in this region has been incomplete^38^.

This GABAergic neuron population in LHb, as characterized here, is dynamically regulated by gonadal functional status, as evidenced by rather pronounced alteration in VGAT mRNA-positive cell density upon castration, and reversal by hormone replacement therapy. The regiospecificity of this effect appears to derive from local conversion of testosterone to estrogen, contributed by convergent aromatase-expressing excitatory inputs to LHbM, also containing neuropeptides or monoamines.

The enzyme aromatase catalyzes the transformation of the androgen testosterone to estrogen. Here, we found aromatase distributed to glutamatergic axon terminals, together with neuropeptides and monoamines. Aromatase activity in these projections may also to be regulated by testosterone levels via AR and to synthesize estradiol. The presence of aromatase at pre-synaptic terminals, allowing highly localized production of estradiol, has been demonstrated at the electron microscope level^65^. Estrogen exerts a synergistic effect on glutamatergic synapses in hippocampus both pre-synaptically and post-synaptically^66^; and estrogen is known to facilitate neuropeptide release^67^. These findings support the organizing actions of androgens as previously suggested^68,69^.

To explore the sex steroid responsivity of these inputs, and of their cell targets in the LHbM, we focused on the well-characterized vasopressinergic input from hypothalamus, the expression of VGAT and ERα in the GERNs themselves, and modulation of motor responses following application of aversive stimuli. Gnx induced a decrease in AVP/aromatase/VGLUT2-positive fibers and in the density of neurons expressing the GERN phenotype in the LHb, and concomitantly, decreased escape behavior during FST and predator exposure tests. HRT restored VP input to LHb, increased the density of cells exhibiting the GERN phenotype in LHb, and restored the propensity for active escape behaviors in both tests. Consistent with these results, GERN cell density was significantly higher in SA male rats, and consequently higher testosterone levels, than SI male rats.

There is evidence that estradiol has enhancing and trophic effects on GABAergic circuits. It has been shown that locally produced, aromatase-dependent, estradiol levels positively correlate with GAD-65 synthesis, thereby supporting GABAergic neurotransmission in cultured neurons^70^. Estrogen has been shown to enhance excitatory neurotransmission by upregulating the expression of glutamate receptors^71^. On the other hand, deletion of ERα expression in GABAergic, but not glutamatergic hippocampal neurons has a pronounced effect on estrogen-dependent behavioral masculinization in the mouse^72^. Moreover, estradiol enhances the release probability of dense core vesicles^73^, which could provide a mechanism of increased excitability due to convergent projections of OR, AVP, DA, and 5-HT.

Neuropeptides have been shown to increase the flexibility of neural circuits by switching the inhibitory/excitatory properties of neural circuits. For instance, it has been demonstrated in *C. elegans* that the neuropeptide INS-6, released in response to large changes in salt concentrations, increase the flexibility of neural circuits, by functionally transforming a sensory neuron in the neural circuit for high salt into a GABAergic interneuron^74^. Also in development, an increase in neural activity per se has been demonstrated to induce a phenotype change from glutamatergic to GABAergic^75^ or in the adult rat hypothalamus, between expression of dopamine or somatostatin in response to short or long photoperiods^76^. Change in neurotransmitters expressed by circuits involved in sensorimotor processes appear to be a common phenomenon by which neural activity, gonadal status or peptidergic neuromodulation combine their actions to fine-tune behavior. The presence of aromatase in the projections onto the lateral habenula, together with the demonstration of estrogen sensitivity in this subpopulation of locally branching GABAergic neurons, support an increased functional inhibition of the habenula depending on diverse neuroendocrine status.

Thus, the GERN of the LHbM, together with their peptidergic and aminergic inputs, represent a node at which several evolutionarily well-conserved motifs—homeostatically sensitive peptide modulation^77–79^, aminergic reward pathways^4,80^, co-regulation of sex steroid synthesis and responsivity^81–83^, and circuit placement that guarantees propagation of peptide, amine, and sex steroid modulation of behavior—all converge.

The physiological state of a network and its level of activity can have a profound effect on neuromodulatory actions on postsynaptic cellular plasticity changes^84^. Our findings provide a concrete example of GABAergic phenotypic switching^85^ and its maintenance as a long-term integrator of inputs from homeostatic and reward-controlling pathways. Modulation of synaptic function within this circuit may be importantly regulated by local (synaptic) conversion of the gonadal steroid testosterone to estrogen via aromatase contained in nerve terminals impingent upon this LHbMC GABAergic cell population.

## Electronic supplementary material

Supplemental Material
