# Supplementary material for: A GABAergic cell type in the lateral habenula links hypothalamic homeostatic and midbrain motivation circuits with sex steroid signaling
Source: Transl Psychiatry. 2018 Feb 26;8:50. doi: 10.1038/s41398-018-0099-5 (PMC5865187; doi:10.1038/s41398-018-0099-5)
Supplement: Supplementary file 1 — Supplemental Material [file 41398_2018_99_MOESM1_ESM.docx]

*Supplemental information (SI)*

A GABAergic cell type in the lateral habenula links hypothalamic homeostatic and midbrain motivation circuits with sex steroid signaling

Limei Zhang^1,2,6^, Vito S. Hernández^1,6^, Jerome D. Swinny^3^, Anil K. Verma^1^, Torsten Giesecke^4^, Andrew C. Emery^2^, Kerim Mutig^4^, Luis M. Garcia-Segura^5^, Lee E. Eiden^2^

^1^Departmento de Fisiología, Facultad de Medicina, Universidad Nacional Autónoma de México, Mexico City, Mexico;

^2^Section on Molecular Neuroscience, National Institute of Mental Health, NIH, Bethesda, USA;

^3^Institute for Biomedical and Biomolecular Sciences, University of Portsmouth, Porthmouth, UK;

Department of Anatomy, Charité-Universitätsmedizin Berlin, Berlin, Germany;

^4^Instituto Cajal, C.S.I.C., Madrid, Spain and CIBERFES, Instituto de Salud Carlos III, Madrid, Spain;

^6^The first two authors contributed equally to this work.

*Running title:*

*LHb GABAergic neurons*

*SI-Figures*


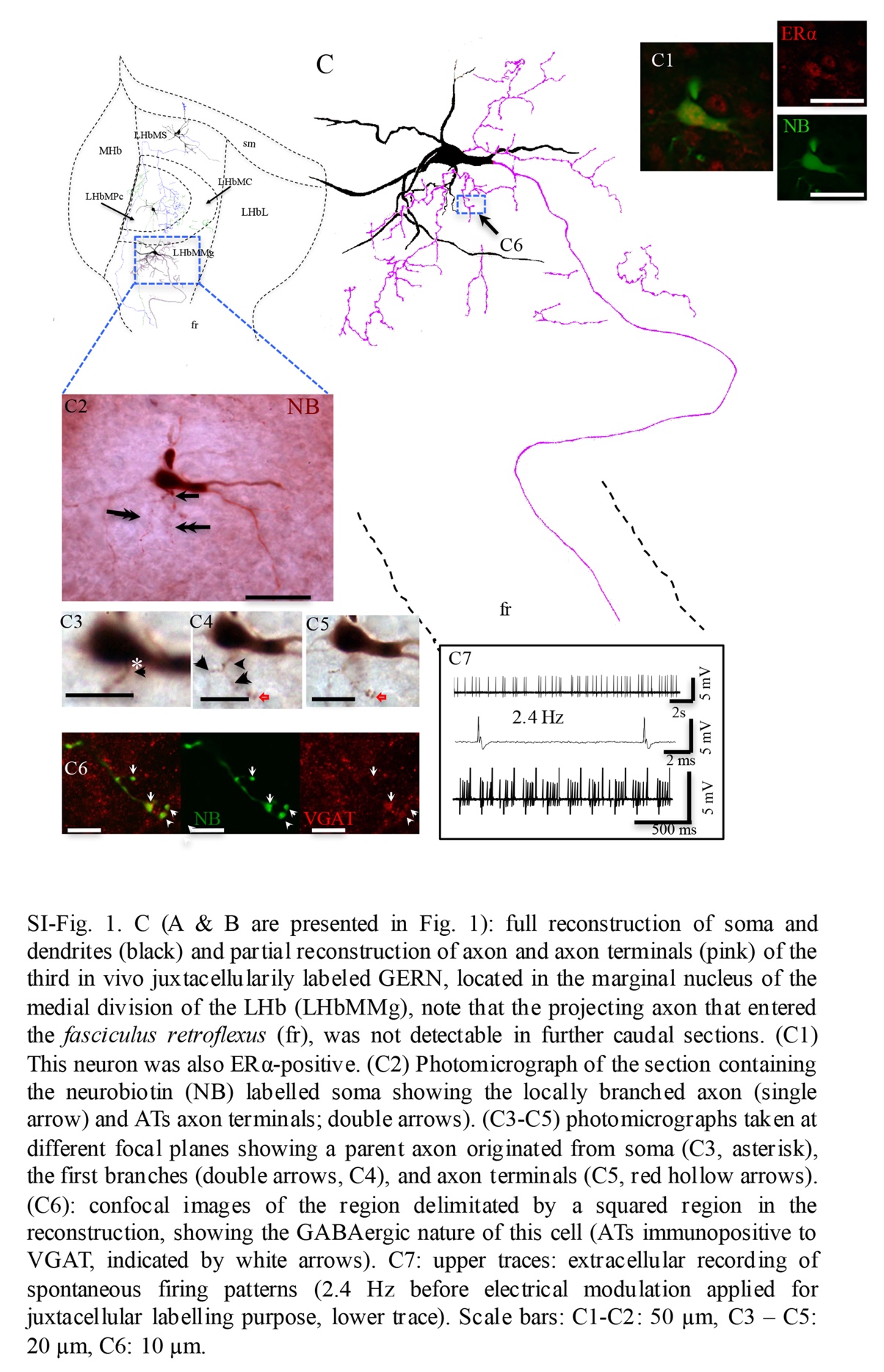


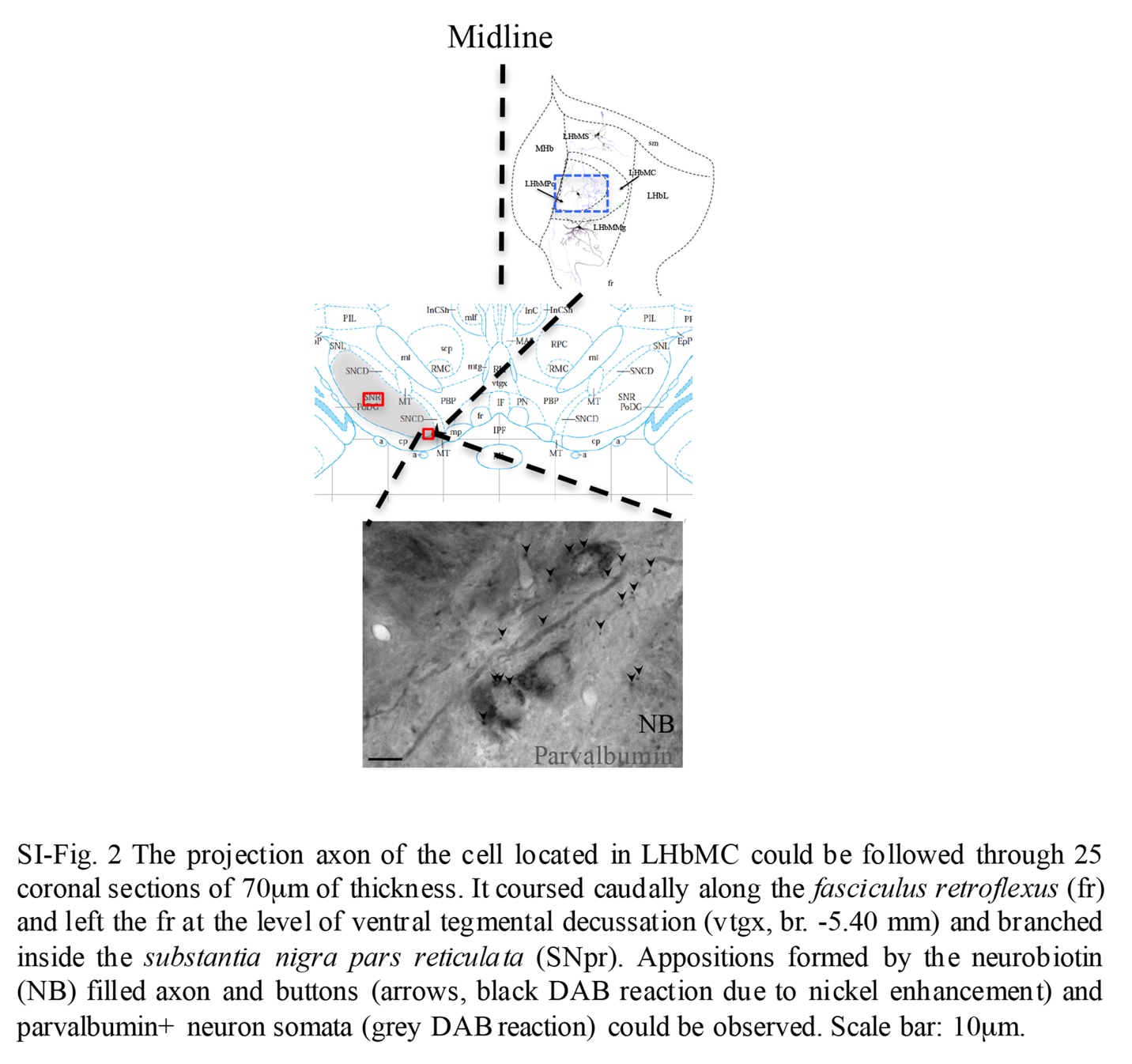


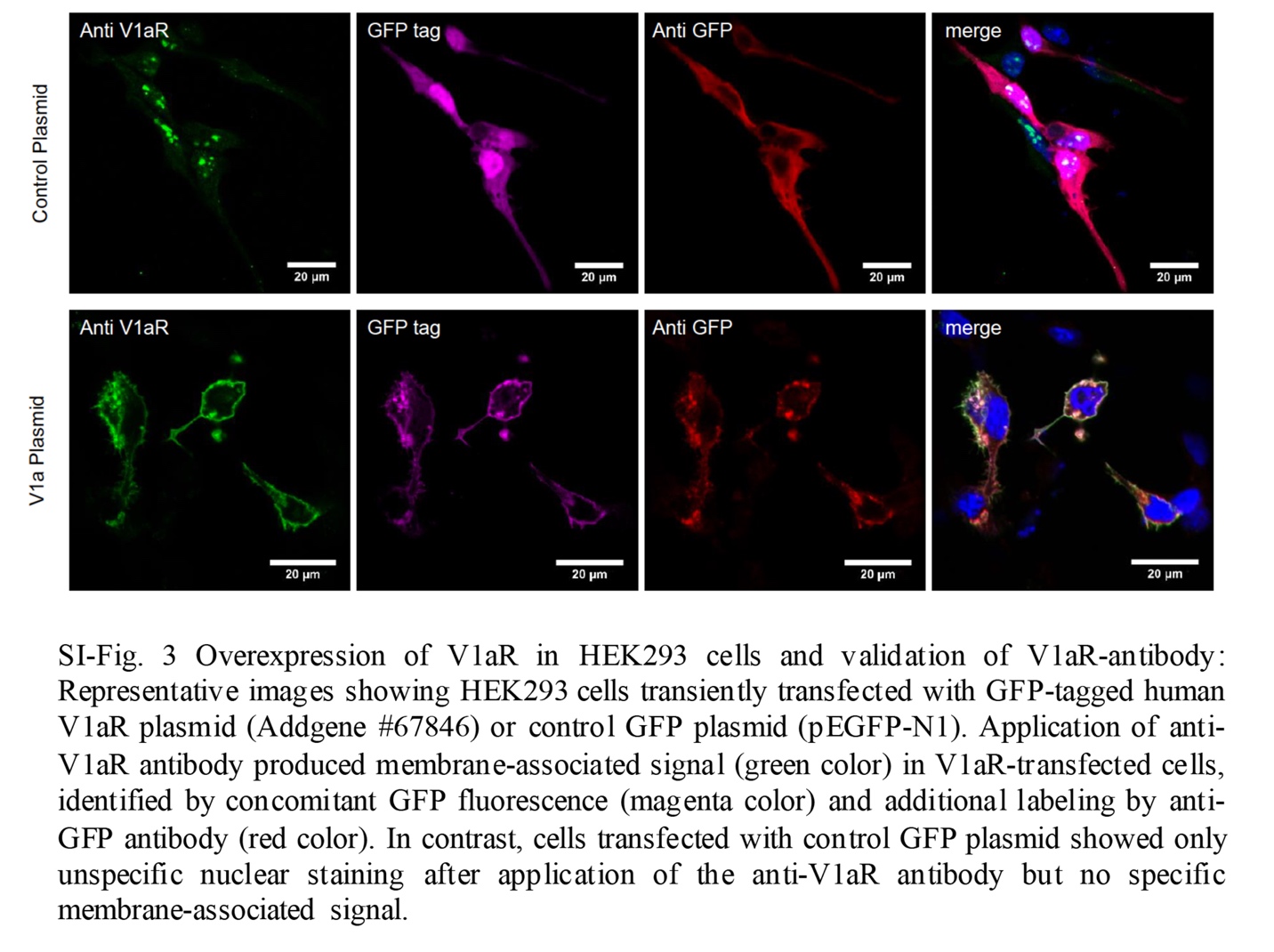

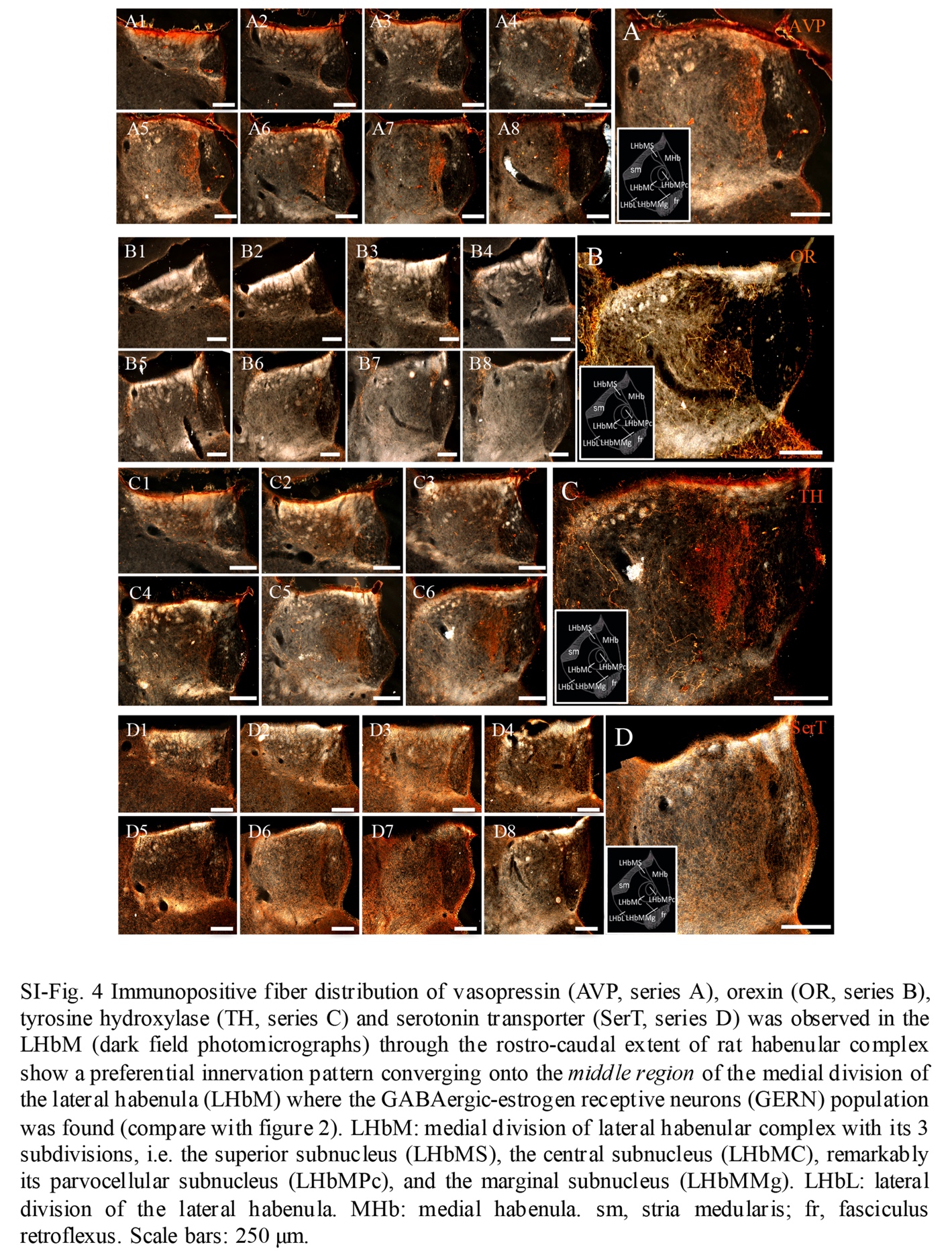


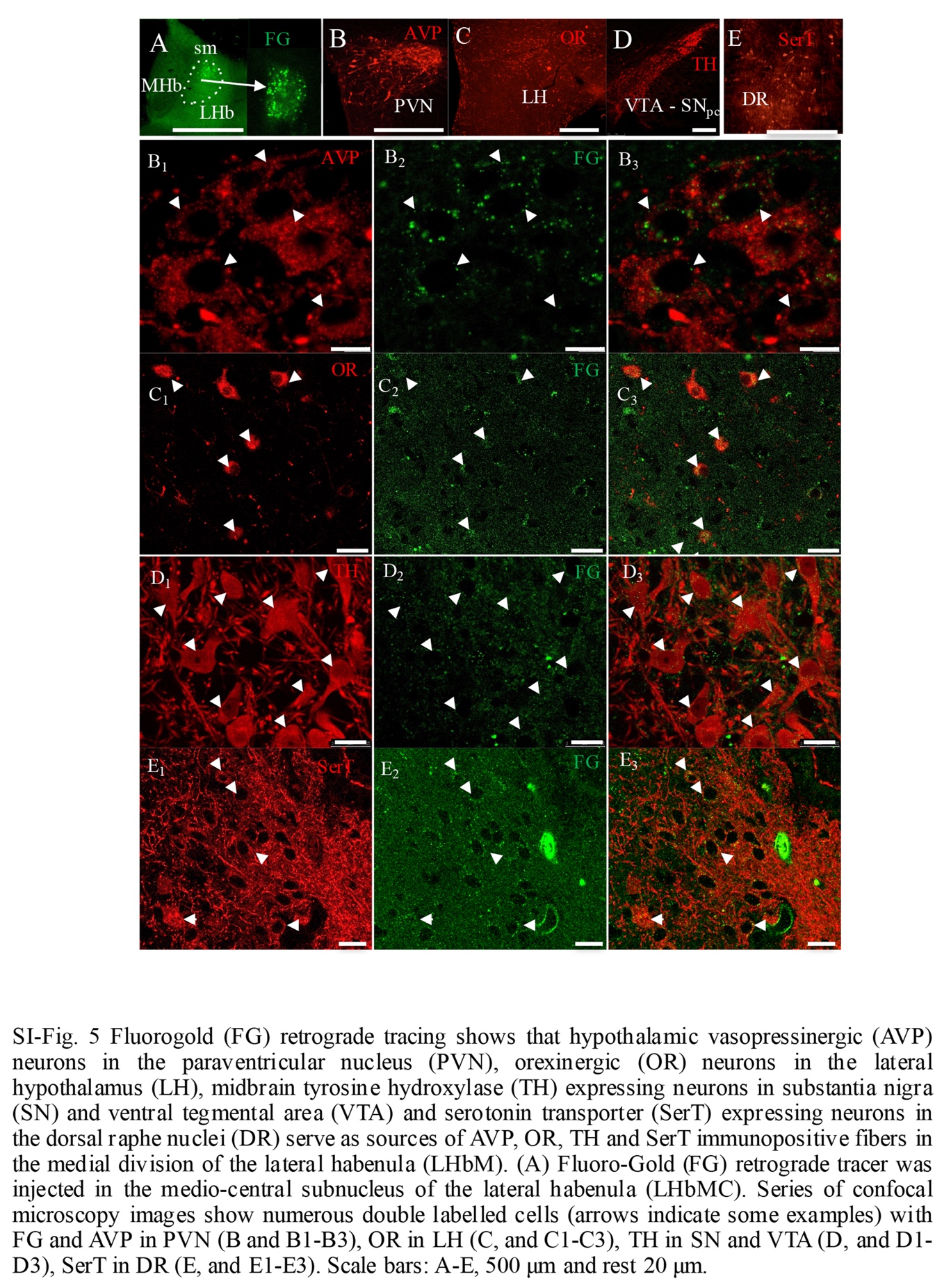


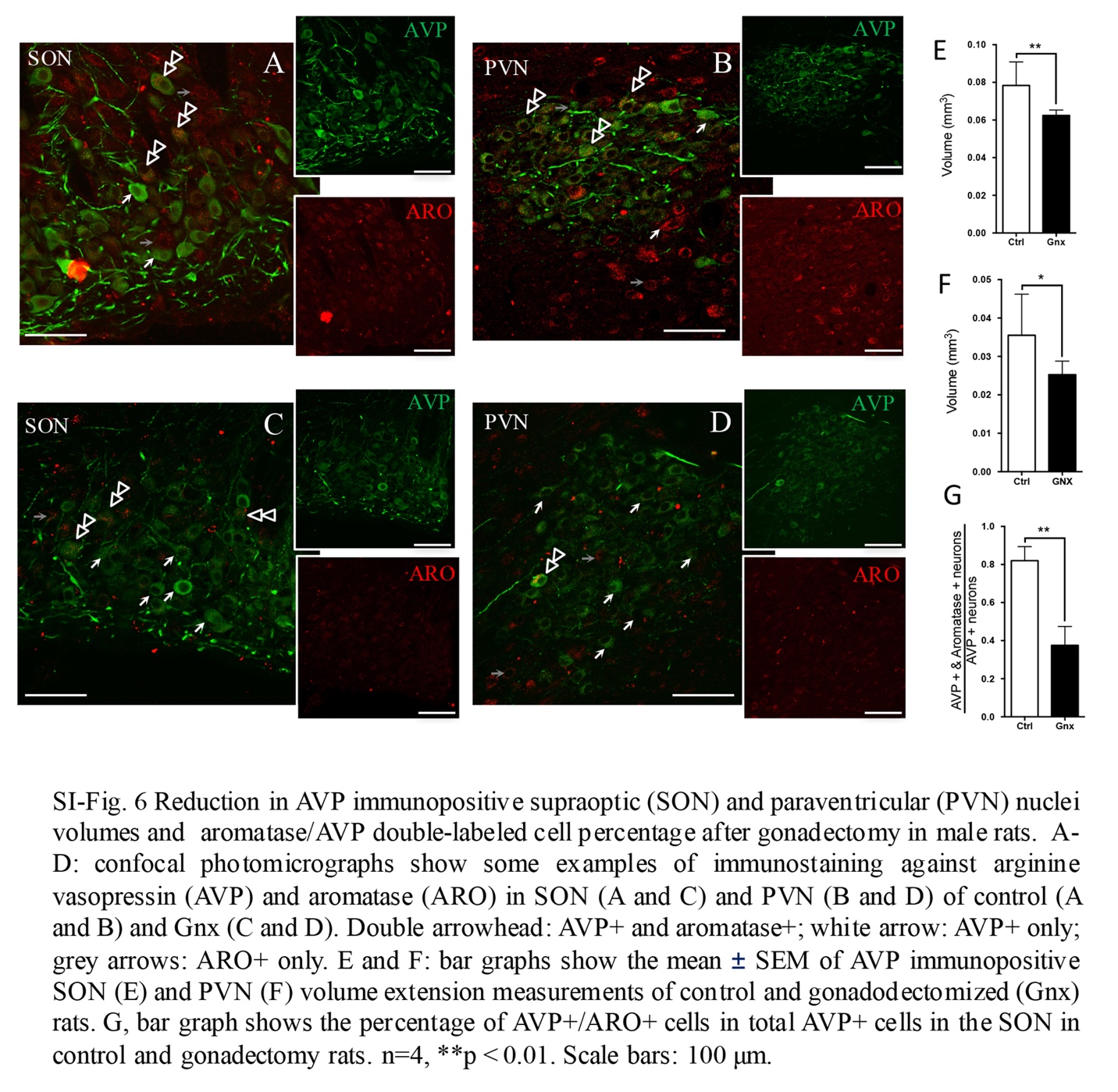


*SI-Experimental Procedures*

Male Wistar rats from local animal breeding facility were used in this study. Approval to conduct the study on animals was obtained from the Ethics Committee of Research Coordination of the Faculty of Medicine, Universidad Nacional Autónoma de México (IDs CIEFM-086-2013 and CIEFM-062-2016).

### 1 Chemicals

Chemicals were obtained from Sigma–Aldrich, St. Louis, MO, USA, if not indicated otherwise. Sources of primary [antibodies](http://www.sciencedirect.com/topics/page/Antibodies) and their dilutions are depicted in [Table 1](http://www.sciencedirect.com/science/article/pii/S0306452212010184#t0005) and Table S1-1.

2 In vivo juxtacellular recording and labelling of single habenular neurons, immunohistochemistry of neurobiotin labelled cells and anatomical reconstruction

For this study, juxtacellular recording and labelling was performed in 48 sexually inactive (SI) male Wistar rats (250 - 300g) according to previous protocols (Leng 1991, Pinault 1996, Tukker, Lasztoczi et al. 2013, Hernandez 2015, Zhang, Hernandez et al. 2016). The induction of anesthesia was achieved using 4% v/v isoflurane (Sofloran Vet, Pisa, Mexico) in O2 and maintained with urethane (1.3g/kg, i.p.; ethyl carbamate; Sigma) and supplemental doses of ketamine (30mg/kg, i. p.; Anesteket, Pisa, Mexico) and xylazine (3 mg/kg, i.p.; Procin, Pisa, Mexico). Wound margins were infiltrated with local anesthetic (lidocaine, Pisa, Mexico). A stereotaxic frame (David Kopf Instruments, CA) was used to fix the animal in place and a homoeothermic heating device (Harvard Apparatus) was used to maintain the rat's temperature at 36 +- 0.5° C. Craniotomy was performed around the coordinates: −3.5 mm posterior from Bregma and 0.5 mm lateral. A long-taper glass electrode (8–15 MΩ) filled with 1% neurobiotin (Vector Laboratories), in 0.15 M NaCl was vertically placed at previously standardized LHbM coordinates (3.6 mm posterior to Bregma, 0.5 mm right/left from midline and 4.3 mm deep) and referenced against a wire implanted subcutaneously in the neck. Neuronal activity was detected, amplified and filtered for single unit recording using differential amplifiers ELC-01MX and DPA-2FL (NPI electronics, GmbH, Tamm, Germany). When a neuron was successfully isolated, it was iontophoretically labeled with neurobiotin using the juxtacellular-labeling method (Pinault 1994). Current pulses of 1–10 nA, at 2.5 Hz, with a 50% duty cycle, were delivered through the recording electrode. The current was gradually increased to induce and maintain entrainment of the activity of the neuron, yielding a higher number of spikes on the current “on” periods. Cells were entrained between 2 and 10 min. After the procedure, the rats were maintained at 35°C during 4–6 h before perfussion, to allow an extensive diffusion of the Neurobiotin through the neurites. Perfusion procedure is described in the section 2.3. Coronal sections cut at 70 µm thickness with a Vibratome (Leica VT 1000S) were stored serially in tissue culture wells in 0.1M PB containing 0.05% NaN_3_. An initial series of one in 2 sections around the electrode tracks was reacted with streptavidin conjugated to Alexa488 (1:1000, Invitrogen), Sections mounted in Vectashield (Vector Laboratories) were assessed under fluorescent microscopy. Sections containing well-labeled somata with observable axon-branching patterns were further processed for VIAAT and ERα immunofluoroscence reaction with corresponding secondary antibodies. After confocal assessment for colocalization, the sections that underwent the streptavin-fluorescence reactions were converted to polymerized 3,3' diaminobenzidine (DAB) horseradish peroxidase (HRP) end product for analysis of neuronal morphology. The detailed methods for anatomical reconstruction have been published previously (Zhang, Hernandez et al. 2016).

3. Immunohistochemistry and indirect immunofluorescence

Unless especified otherwise, we used sexually active (SA) Wistar (300g - 400g, b.w.) rats from local animal facility. In the experiments where the effect of gonadal steroids was evaluated we used rats that were gonadectomized or gonadectomized and treated for two months before the perfussion. Rats under all the above mentioned conditions, received an intraperitoneal overdose of sodium pentobarbital (63 mg/kg, Sedalpharma, México) and were perfused transaortically with 0.9% saline followed by cold fixative containing 4% w/v of paraformaldehyde in 0.1 M sodium phosphate buffer (PB, pH 7.4) plus 15% v/v saturated picric acid for 15 min (for immunoreaction using antibody against GABA, the fixative was added 0.1% of glutaraldehyde additionally). Brains were immediately removed, blocked, and then thoroughly rinsed with PB. Brains were sectioned soon after perfusion using a Leica VT 1000S vibratome. Freshly-cut freely-floating sections were blocked with 20% normal donkey serum (NDS) in Tris-buffered (0.05 M, pH 7.4) saline (0.9%) plus 0.3% of Triton X-100 (TBST) for 1 h at room temperature and incubated with the primary antibodies listed in Table 1 (for antibody specificity see supplementary information Table S1). For light microscopy immunohistochemistry, Vectastain Elite ABC Kit (Vector Labs, Burlingame, CA) followed by DAB-peroxidase reaction was done while for immunofluorescence reactions, sections were incubated with the corresponding fluorochrome-conjugated secondary antibodies.

4 Anti-V1aR antibody (AVPR1A) production and validation

The anti-V1aR antibody (AVPR1A) was produced using the specific peptide (NH2-CKDSPKSSKSIRFIPVST-COOH) for immunization of rabbits. The resulting antisera were obtained after 6 months of immunization, followed by affinity purification (Pineda, Berlin, Germany custom made for Mutig Group Charité). The validation of the antibody was verified by immunofluorescence labeling of HEK293 cells, transfected with GFP-tagged human V1aR plasmid (Addgene plasmid 67846) vs. Control Plasmid (pEGFP-N1). The HEK293 cells were cultivated in cell culture dishes with cover slips in DMEM medium (PAN-Biotech) at 37°C, 95% humidity and 5% CO_2_ and were transfected with the respective plasmids using JetPEI transfection reagent (Polyplus) followed by Incubation for 48 hours at 37°C. Cover Slips were obtained for immunofluorescence analysis, washed with PBS, fixated with 4% paraformaldehyde/PBS for 10 min, permeabilized, with 0,1% Triton X-100 (Merck) in TBS and blocked with 5% BSA ( bovine serum albumin, SERVA) in TBS for 30 min. All antibodies were diluted in 5% BSA/TBS. Incubation of the AVPR1A was 30 min at room temperature (RT) followed by overnight incubation at 4°C. All other antibodies (GFP and secondary antibodies) were incubated at RT for 2 hours. Pictures were taken with Zeiss LSM 5 exciter confocal microscope and processed with ZEISS ZEN-Imaging Software.

5 Fluoro-gold retrograde tracing

Anaesthesia was induced and maintained with ketamine (100mg/kg, IP) and xylazine (10mg/kg, IP, Procin, Pisa, Mexico). Rats were fixed in a stereotaxic apparatus and were injected in the medial Lateral habenula LHbM, [Bregma -3.5 mm, medio-lateral 0.5 mm, dorsoventral 4.7 mm ([Paxinos and Watson, 2006](#ENREF_13))] with the retrograde tracer Fluoro-Gold (FG, Fluorochrome, LLC, Denver, Colorado, 80218 USA), dissolved 1% in 0.2 M sodium cacodylate buffer (pH 7.5). The FG was delivered iontophoretically using an iontophoresis pump (Value Kation Sci VAB-500) through a stereotaxically positioned glass micropipette with an inner tip diameter of approximately 40 μm, by applying current pulses of 0.1 μA, at 0.2Hz, with a 50% duty cycle, for 20 min. The micropipette was left in place for an additional 10 min to prevent backflow of the tracer up the injection track after each injection. After completing the surgery, rats received 0.4 mg/kg *i.p.* Ketorolac (Apotex, Mexico) and 50 mg/kg *i.p* ceftriaxone (Kendric, Mexico) as analgesic/anti-inflammatory and antibiotic agents, respectively. Three to four weeks after the FG injections, the rats were perfused as previously described (Zhang and Hernandez 2013). Coronal and sagittal sections of 70 μm were obtained, and VP IHC was performed to evaluate if the SON and PVN VP+ neurons were labelled with FG. Observations were made under light (Nikon ECLIPSE 50i with B-2A long-pass emission filter) and confocal microscopy (Leica TCS-SP5).

6 RNAscope ISH assays

Two SA rats (300g - 400g) were deeply anesthetized and decapitated using small animal guillotine (Kent Scientific corp.). Brains were removed and rapidly frozen through carefully burying them in dry-ice powder. The fresh-frozen tissue sections (12 μm thick) were aquired with the aid of a cryostat Leica CM-1520 and mounted on positively charged glass slides (Fisher Scientific, Pittsburgh, PA). The RNA probes for in situ hybridization used in this study to identify the genes Slc32a1, Esr1, Hcrtr2 were were designed and provided by Advanced Cell Diagnostics (Hayward, CA, Rn-Slc32a1, Cat No. 424541-C3; Rn-Esr1, Cat. No. 317151; Rn-Hcrtr2-C2, Cat. No. 484571, Rn-Slc17a6 Cat. No. 317011-C3, Rn-Gad1-C2, Cat No. 316401-C2, Rn-Gad1-C2, Cat No. 435801-C2). All staining steps were performed following RNAscope protocol for Multiplex fresh frozen sections. Stained slides were coverslipped with fluorescent mounting medium (ProLong Gold Antifade Reagent P36930; Life Technologies) and examined with a confocal microscope (Leica TCS-SP5) at 63x magnification using the manufacturer-provided software.

7 Gonadectomy and hormone replacement therapy

Juvenile male rats of post-natal day 35 were used. Under anesthesia with ketamine (100mg/kg, IP) and xylazine (10mg/kg, IP, Procin, Pisa, Mexico), a small surgical incision was made in the center of the scrotum. The testicles and spermatic cord were exposed through the surgical wound, then the spermatic cord was cauterized and the testicles removed. The incision was closed with nylon 3-0 sutures and rats treated with ketorolac and ceftriaxone during the post-operative period. Monthly s.c. injections of Sustanon (250 mg/kg body weight; Sustanon 250 is a long-acting mixture of testosterone esters – testosterone propionate (20%), testosterone phenylpropionate (40%) and testosterone isocaproate (40%)) (Organon Mexicana, CdMx, Mexico) were given. Rats were housed in pairs for 2 months before the experiment.

8. Live cat exposure and behavioral scoring

The behavior test was performed during the early activity period of the rats (dark period). Experimental subjects housed three per cage, were divided into two groups: the intact group and the gonadectomized (GNX). For innate fear assessment, each rat was placed individually in a grid cage (28.5 × 21 × 30 cm), so the rat could climb. The cage was placed inside a larger ventilated clear plastic chamber (60 × 80 × 40 cm), where a male adult cat was then introduced. The cat was tamed and castrated, about 5 kg of body weight. The cat was mostly kept quiet/immobile during the experiment. One advantage of this arrangement is that the rats were exposed to physiologically relevant stimuli—a live predator's odor, appearance and breathing sounds, which were relatively constant for all the experimental subjects. Each rat remained in the chamber described above for a single period of 10 min. Once the time of exposure was completed, they were returned to their home cage.

Relevant behaviors were quantified offline by giving one of the six scores every 5 s: (1) “Freezing” was assigned to the behavior of immobility for more than 2 s with pilo-erection; (2) “Climbing”: when rats climbed the internal mesh cage using limbs trying to escape from the top door; (3) “Rearing,” when rats were rearing still, sniffing with short head rotations; (4) “Displacement,” when the rats were walking, trotting or running; (5) “Orientation”: when the four limbs of the rats were still with head extension, rotation; (6) “Grooming”: when rats groomed themselves (nose, head, face, eyes, and body) with their paws, using very short movements. “Active Escaping” measured in the study included the behaviors 2–4.

9 Forced Swimming Test

In this test, intact rats with or without water and Food deprivation (WFD) and GNX rats with or without hormone replacement treatment (HRT) were placed into 45 cm height × 30 cm diameter Plexiglas cylinders, filled with water at 25°C, up to a height of 25 cm, and their behavior was recorded over a 6-min test period. The behavior was evaluated offline using the criteria described previously (Detke et al., 1995; Zhang et al., 2008). The observers scored the swimming behavior of the rat every 5s, and if there was cessation of spatial displacement with or without minor

10 Statistical Analysis

Quantitative results were expressed as mean ± SEM. Groups were tested for normality with a D'Agostino and Pearson test, then differences between groups were calculated by Student t-test or analysis of variance followed by the Bonferroni test, using Prism (GraphPad Software, San Diego, CA, USA). Differences were considered statistically significant at (*P < 0.05, **P < 0.01, and ***P < 0.001).

References:

Hernandez, V. H., Vazquez-Juarez, E., Marquez M.M., Jauregui Huerta F., Barrio, R. A. and Zhang, L (2015). "Extra-neurohypophyseal axonal projections from individual vasopressin-containing magnocellular neurons in rat hypothalamus." Frontier in Neuroanatomy **9:130**.

Leng, G. a. D., R. E. J. (1991). Functional identification of magnocellular neuroendocrine neurons. Neuroendocrine Research Methods. B. Greenstein, Harwood Academics Publishers GmbH**:** 769–791.

Pinault, D. (1994). "Golgi-like labeling of a single neuron recorded extracellularly." Neurosci Lett **170**(2): 255-260.

Pinault, D. (1996). "A novel single-cell staining procedure performed in vivo under electrophysiological control: morpho-functional features of juxtacellularly labeled thalamic cells and other central neurons with biocytin or Neurobiotin." J Neurosci Methods **65**(2): 113-136.

Tukker, J. J., B. Lasztoczi, L. Katona, J. D. Roberts, E. K. Pissadaki, Y. Dalezios, L. Marton, L. Zhang, T. Klausberger and P. Somogyi (2013). "Distinct dendritic arborization and in vivo firing patterns of parvalbumin-expressing basket cells in the hippocampal area CA3." J Neurosci **33**(16): 6809-6825.

Zhang, L. and V. S. Hernandez (2013). "Synaptic innervation to rat hippocampus by vasopressin-immuno-positive fibres from the hypothalamic supraoptic and paraventricular nuclei." Neuroscience **228**: 139-162.

Zhang, L., V. S. Hernandez, E. Vazquez-Juarez, F. K. Chay and R. A. Barrio (2016). "Thirst Is Associated with Suppression of Habenula Output and Active Stress Coping: Is there a Role for a Non-canonical Vasopressin-Glutamate Pathway?" Front Neural Circuits **10**: 13.

| Molecule | | Host species | Dilution | Source | Source code | Antibody Specificity Information |
| --- | --- | --- | --- | --- | --- | --- |
| [Arg8 ]-vasopressin | | rabbit | 1:5000 | Peninsula-Bachem Americas, Inc., CA, USA. (www.bachem.com) | T-4563 | See references (Taylor, McCarthy et al. 2008, Zhang and Hernandez 2013) |
| [Arg8 ]-vasopressin | | rabbit | 1:2000 | Gift from Prof. R.M. Buijs, Instituto de Investigaciones Biomédicas, Universidad Nacional Autónoma de México, UNAM | -- | See reference (Buijs, Pool et al. 1989) |
| Tyrosine Hydroxylase | | sheep | 1:2000 | EMD Millipore Corporation, Billerica, MA, USA (www.millipore.com) | AB-1542 | See references (Haycock and Waymire 1982, Kaufling, Veinante et al. 2009, Geerling, Shin et al. 2010, Li, Shi et al. 2014, Garcia-Aviles, Albert-Gasco et al. 2015) |
| Serotonin Transporter (SerT) | | goat | 1:2000 | Santa Cruz Biotechnology, Dallas, Texas U.S.A (www.scbt.com) | SC-1458 | See references (Pickel and Chan 1999, Ebner, Singewald et al. 2008, Tata and Yamamoto 2008) |
| Hypocretin/orexin (OR) | | rabbit | 1:2000 (LM, EM) | Gift from Prof. Anthony van den Pol (Yale School of Medicine, Yale Cancer Center: Virus and Other Infection-associated Cancers) | Hypocretin/orexin (OR) | See references (Peyron, Tighe et al. 1998, van den Pol 1999) |
| Vesicular glutamate transporter 2 | | guinea pig | 1:1000 | Frontier Institute Co., Ltd., Hokkaido , Japan (www.frontier-institute.com) | GP-AF240-1 | See reference (Miyazaki, Fukaya et al. 2003, Zhang, Qi et al. 2015) |
| Vesicular inhibitory amino acid transporter (VIAAT) | | rabbit | 1:2000 | Lee E Eiden, Section on Molecular Neuroscience, National Institute of Mental Health, NIH, Bethesda, USA | -- | See reference (Weihe and Eiden 2000) |
| Gamma-aminobutyric Acid (GABA) | | mouse | 1:1000 | Sigma-Aldrich Corporation, MO, USA (www.sigmaaldrich.com) | A0310 | See references (Sloviter, Ali-Akbarian et al. 2001, Omelchenko and Sesack 2006, Gonchar, Wang et al. 2007) |
| Glutamic acid decarboxylase 65 kDa isoform (GAD 65) | | mouse | 1:2000 | EMD Millipore Corporation, Billerica, MA, USA (www.millipore.com) | MAB351 | See references (Kaufling, Veinante et al. 2009, Rostkowski, Teppen et al. 2009, Cserep, Szonyi et al. 2011, Stensrud, Puchades et al. 2014) |
| Glutamic acid decarboxylase 67 kDa isoform (GAD 67) | | mouse | 1:2000 | EMD Millipore Corporation, Billerica, MA, USA (www.millipore.com) | MAB5406 | See references (Arime, Kasahara et al. 2012, Boulland and Chaudhry 2012, Margolis, Toy et al. 2012, Tognini, Manno et al. 2012, Wen, Nguyen et al. 2014) |
| Parvalbumin | | mouse | 1:5000 (LM) | Swant, Marly, Switzerland, (www.swant.com) | Cat. 235 | See references (Celio and Heizmann 1981, Wang, Cheng et al. 2015) |
| P450 Aromatase | | rabbit | 1:2000 (LM) | Luis Miguel García-Segura, Instituto Cajal, C.S.I.C., Madrid, Spain | -- | See references (Garcia-Segura, Wozniak et al. 1999, Pietranera, Bellini et al. 2011, Cisternas, Tome et al. 2015, Pietranera, Correa et al. 2016) |
| Estrogen receptor alpha | | rabbit | 1:2000 | Santa Cruz laboratories.  Dallas, Texas U.S.A (www.scbt.com) | SC - 542 | See references (Reboucas, Leal et al. 2016, Sa and Fonseca 2017) |
| Androgen receptor | | rabbit | 1:2000 | Santa Cruz laboratories, Dallas, Texas U.S.A (www.scbt.com) | SC - 816 | See references (Normandin and Murphy 2008, Bender, Zhou et al. 2017) |
| Dopamine receptor 5 | | rabbit | 1:1000 | Alomone Labs. Jerusalem, Israel, (http://www.alomone.com) | ADR-005 | See reference: (Chen, Hong et al. 2014) |
| Serotonin receptor 5-HTR2A | | mouse | 1:200 | BD pharmigen, | Cat. 556326 | See reference (McDonald, & Mascagni (2007) |
| [Arg8 ]-vasopressin receptor 1A (AVPR1A) | rabbit | 1:1000 | Kerim Mutig and Torsten Giesecke  Institute of Vegetative Anatomy, Charité – Universitätsmedizin Berlin, Germany | - | See SI method and figure 3 for details. | |
| Green fluorescent protein (GFP) | mouse | 1:1500 | Abcam, Cambridge, UK | Ab-291-50 | See reference (Brandt N et al. 2008) | |

*SI-* *Table Antibodies information*

**References**

Arime, Y., Y. Kasahara, F. S. Hall, G. R. Uhl and I. Sora (2012). "Cortico-subcortical neuromodulation involved in the amelioration of prepulse inhibition deficits in dopamine transporter knockout mice." Neuropsychopharmacology **37**(11): 2522-2530.

Bender, R. A., L. Zhou, R. Vierk, N. Brandt, A. Keller, C. E. Gee, M. K. Schafer and G. M. Rune (2017). "Sex-Dependent Regulation of Aromatase-Mediated Synaptic Plasticity in the Basolateral Amygdala." J Neurosci **37**(6): 1532-1545.

Boulland, J. L. and F. A. Chaudhry (2012). "Ontogenetic changes in the distribution of the vesicular GABA transporter VGAT correlate with the excitation/inhibition shift of GABA action." Neurochem Int **61**(4): 506-516.

Buijs, R., C. Pool, J. Van Heerikhuize, A. Sluiter, P. Van der Sluis, M. Ramkena, T. Van der Woude and E. Van der Beek (1989). "Antibodies to small transmitter molecules and peptides: production and application of antibodies to dopamine, serotonin, GABA, vasopressin, vasoactive intestinal peptide, neuropeptide Y, somatostatine and substance P." Biomedical research **10**(supplement 3): 213-221.

Celio, M. R. and C. W. Heizmann (1981). "Calcium-binding protein parvalbumin as a neuronal marker." Nature **293**(5830): 300-302.

Chang, W., H. Kanda, R. Ikeda, J. Ling, J. J. DeBerry and J. G. Gu (2016). "Merkel disc is a serotonergic synapse in the epidermis for transmitting tactile signals in mammals." Proc Natl Acad Sci U S A **113**(37): E5491-5500.

Chen, Y., F. Hong, H. Chen, R. F. Fan, X. L. Zhang, Y. Zhang and J. X. Zhu (2014). "Distinctive expression and cellular distribution of dopamine receptors in the pancreatic islets of rats." Cell Tissue Res **357**(3): 597-606.

Cisternas, C. D., K. Tome, X. E. Caeiro, F. M. Dadam, L. M. Garcia-Segura and M. J. Cambiasso (2015). "Sex chromosome complement determines sex differences in aromatase expression and regulation in the stria terminalis and anterior amygdala of the developing mouse brain." Mol Cell Endocrinol **414**: 99-110.

Cserep, C., A. Szonyi, J. M. Veres, B. Nemeth, E. Szabadits, J. de Vente, N. Hajos, T. F. Freund and G. Nyiri (2011). "Nitric oxide signaling modulates synaptic transmission during early postnatal development." Cereb Cortex **21**(9): 2065-2074.

Ebner, K., G. M. Singewald, N. Whittle, F. Ferraguti and N. Singewald (2008). "Neurokinin 1 receptor antagonism promotes active stress coping via enhanced septal 5-HT transmission." Neuropsychopharmacology **33**(8): 1929-1941.

Garcia-Aviles, A., H. Albert-Gasco, I. Arnal-Vicente, E. Elhajj, J. Sanjuan-Arias, A. M. Sanchez-Perez and F. Olucha-Bordonau (2015). "Acute oral administration of low doses of methylphenidate targets calretinin neurons in the rat septal area." Front Neuroanat **9**: 33.

Garcia-Segura, L. M., A. Wozniak, I. Azcoitia, J. R. Rodriguez, R. E. Hutchison and J. B. Hutchison (1999). "Aromatase expression by astrocytes after brain injury: implications for local estrogen formation in brain repair." Neuroscience **89**(2): 567-578.

Geerling, J. C., J. W. Shin, P. C. Chimenti and A. D. Loewy (2010). "Paraventricular hypothalamic nucleus: axonal projections to the brainstem." J Comp Neurol **518**(9): 1460-1499.

Gonchar, Y., Q. Wang and A. Burkhalter (2007). "Multiple distinct subtypes of GABAergic neurons in mouse visual cortex identified by triple immunostaining." Front Neuroanat **1**: 3.

Haycock, J. W. and J. C. Waymire (1982). "Activating antibodies to tyrosine hydroxylase." J Biol Chem **257**(16): 9416-9423.

Hernandez, V. H., Vazquez-Juarez, E., Marquez M.M., Jauregui Huerta F., Barrio, R. A. and Zhang, L (2015). "Extra-neurohypophyseal axonal projections from individual vasopressin-containing magnocellular neurons in rat hypothalamus." Frontier in Neuroanatomy **9:130**.

Kaufling, J., P. Veinante, S. A. Pawlowski, M. J. Freund-Mercier and M. Barrot (2009). "Afferents to the GABAergic tail of the ventral tegmental area in the rat." J Comp Neurol **513**(6): 597-621.

Leng, G. a. D., R. E. J. (1991). Functional identification of magnocellular neuroendocrine neurons. Neuroendocrine Research Methods. B. Greenstein, Harwood Academics Publishers GmbH**:** 769–791.

Li, S., Y. Shi and G. J. Kirouac (2014). "The hypothalamus and periaqueductal gray are the sources of dopamine fibers in the paraventricular nucleus of the thalamus in the rat." Front Neuroanat **8**: 136.

McDonald, A. J., & Mascagni, F. (2007). Neuronal Localization of 5-HT2A Receptor Immunoreactivity in the Rat Basolateral Amygdala. Neuroscience, 146(1), 306–320. http://doi.org/10.1016/j.neuroscience.2007.01.047

Margolis, E. B., B. Toy, P. Himmels, M. Morales and H. L. Fields (2012). "Identification of rat ventral tegmental area GABAergic neurons." PLoS One **7**(7): e42365.

Miyazaki, T., M. Fukaya, H. Shimizu and M. Watanabe (2003). "Subtype switching of vesicular glutamate transporters at parallel fibre-Purkinje cell synapses in developing mouse cerebellum." Eur J Neurosci **17**(12): 2563-2572.

Normandin, J. J. and A. Z. Murphy (2008). "Nucleus paragigantocellularis afferents in male and female rats: organization, gonadal steroid receptor expression, and activation during sexual behavior." J Comp Neurol **508**(5): 771-794.

Omelchenko, N. and S. R. Sesack (2006). "Cholinergic axons in the rat ventral tegmental area synapse preferentially onto mesoaccumbens dopamine neurons." J Comp Neurol **494**(6): 863-875.

Peyron, C., D. K. Tighe, A. N. van den Pol, L. de Lecea, H. C. Heller, J. G. Sutcliffe and T. S. Kilduff (1998). "Neurons containing hypocretin (orexin) project to multiple neuronal systems." J Neurosci **18**(23): 9996-10015.

Pickel, V. M. and J. Chan (1999). "Ultrastructural localization of the serotonin transporter in limbic and motor compartments of the nucleus accumbens." J Neurosci **19**(17): 7356-7366.

Pietranera, L., M. J. Bellini, M. A. Arevalo, R. Goya, M. E. Brocca, L. M. Garcia-Segura and A. F. De Nicola (2011). "Increased aromatase expression in the hippocampus of spontaneously hypertensive rats: effects of estradiol administration." Neuroscience **174**: 151-159.

Pietranera, L., J. Correa, M. E. Brocca, P. Roig, A. Lima, N. Di Giorgio, L. M. Garcia-Segura and A. F. De Nicola (2016). "Selective Oestrogen Receptor Agonists Rescued Hippocampus Parameters in Male Spontaneously Hypertensive Rats." J Neuroendocrinol **28**(10).

Pinault, D. (1994). "Golgi-like labeling of a single neuron recorded extracellularly." Neurosci Lett **170**(2): 255-260.

Pinault, D. (1996). "A novel single-cell staining procedure performed in vivo under electrophysiological control: morpho-functional features of juxtacellularly labeled thalamic cells and other central neurons with biocytin or Neurobiotin." J Neurosci Methods **65**(2): 113-136.

Reboucas, E. C. C., S. Leal, S. M. Silva and S. I. Sa (2016). "Changes in the female arcuate nucleus morphology and neurochemistry after chronic ethanol consumption and long-term withdrawal." J Chem Neuroanat **77**: 30-40.

Rostkowski, A. B., T. L. Teppen, D. A. Peterson and J. H. Urban (2009). "Cell-specific expression of neuropeptide Y Y1 receptor immunoreactivity in the rat basolateral amygdala." J Comp Neurol **517**(2): 166-176.

Sa, S. I. and B. M. Fonseca (2017). "Dynamics of progesterone and estrogen receptor alpha in the ventromedial hypothalamus." J Endocrinol **233**(2): 197-207.

Sloviter, R. S., L. Ali-Akbarian, K. D. Horvath and K. A. Menkens (2001). "Substance P receptor expression by inhibitory interneurons of the rat hippocampus: enhanced detection using improved immunocytochemical methods for the preservation and colocalization of GABA and other neuronal markers." J Comp Neurol **430**(3): 283-305.

Stensrud, M. J., M. Puchades and V. Gundersen (2014). "GABA is localized in dopaminergic synaptic vesicles in the rodent striatum." Brain Struct Funct **219**(6): 1901-1912.

Tata, D. A. and B. K. Yamamoto (2008). "Chronic stress enhances methamphetamine-induced extracellular glutamate and excitotoxicity in the rat striatum." Synapse **62**(5): 325-336.

Taylor, A. C., J. J. McCarthy and S. D. Stocker (2008). "Mice lacking the transient receptor vanilloid potential 1 channel display normal thirst responses and central Fos activation to hypernatremia." Am J Physiol Regul Integr Comp Physiol **294**(4): R1285-1293.

Tognini, P., I. Manno, J. Bonaccorsi, M. C. Cenni, A. Sale and L. Maffei (2012). "Environmental enrichment promotes plasticity and visual acuity recovery in adult monocular amblyopic rats." PLoS One **7**(4): e34815.

Tukker, J. J., B. Lasztoczi, L. Katona, J. D. Roberts, E. K. Pissadaki, Y. Dalezios, L. Marton, L. Zhang, T. Klausberger and P. Somogyi (2013). "Distinct dendritic arborization and in vivo firing patterns of parvalbumin-expressing basket cells in the hippocampal area CA3." J Neurosci **33**(16): 6809-6825.

van den Pol, A. N. (1999). "Hypothalamic hypocretin (orexin): robust innervation of the spinal cord." J Neurosci **19**(8): 3171-3182.

Wang, W. C., C. F. Cheng and M. L. Tsaur (2015). "Immunohistochemical localization of DPP10 in rat brain supports the existence of a Kv4/KChIP/DPPL ternary complex in neurons." J Comp Neurol **523**(4): 608-628.

Weihe, E. and L. E. Eiden (2000). "Chemical neuroanatomy of the vesicular amine transporters." FASEB J **14**(15): 2435-2449.

Wen, Z., H. N. Nguyen, Z. Guo, M. A. Lalli, X. Wang, Y. Su, N. S. Kim, K. J. Yoon, J. Shin, C. Zhang, G. Makri, D. Nauen, H. Yu, E. Guzman, C. H. Chiang, N. Yoritomo, K. Kaibuchi, J. Zou, K. M. Christian, L. Cheng, C. A. Ross, R. L. Margolis, G. Chen, K. S. Kosik, H. Song and G. L. Ming (2014). "Synaptic dysregulation in a human iPS cell model of mental disorders." Nature **515**(7527): 414-418.

Zhang, L. and V. S. Hernandez (2013). "Synaptic innervation to rat hippocampus by vasopressin-immuno-positive fibres from the hypothalamic supraoptic and paraventricular nuclei." Neuroscience **228**: 139-162.

Zhang, L., V. S. Hernandez, E. Vazquez-Juarez, F. K. Chay and R. A. Barrio (2016). "Thirst Is Associated with Suppression of Habenula Output and Active Stress Coping: Is there a Role for a Non-canonical Vasopressin-Glutamate Pathway?" Front Neural Circuits **10**: 13.

Zhang, S., J. Qi, X. Li, H. L. Wang, J. P. Britt, A. F. Hoffman, A. Bonci, C. R. Lupica and M. Morales (2015). "Dopaminergic and glutamatergic microdomains in a subset of rodent mesoaccumbens axons." Nat Neurosci **18**(3): 386-392.
